# Sinorhizobium meliloti Functions Required for Resistance to Antimicrobial NCR Peptides and Bacteroid Differentiation

**DOI:** 10.1128/mBio.00895-21

**Published:** 2021-07-27

**Authors:** Quentin Nicoud, Quentin Barrière, Nicolas Busset, Sara Dendene, Dmitrii Travin, Mickaël Bourge, Romain Le Bars, Claire Boulogne, Marie Lecroël, Sándor Jenei, Atilla Kereszt, Eva Kondorosi, Emanuele G. Biondi, Tatiana Timchenko, Benoît Alunni, Peter Mergaert

**Affiliations:** a Université Paris-Saclay, CEA, CNRS, Institute for Integrative Biology of the Cell (I2BC), Gif-sur-Yvette, France; b Center of Life Sciences, Skolkovo Institute of Science and Technology, Moscow, Russia; c Institute of Plant Biology, Biological Research Centre, Szeged, Hungary; University of Connecticut

**Keywords:** *Sinorhizobium meliloti*, antimicrobial peptides, mechanisms of resistance, nitrogen fixation, symbiosis

## Abstract

Legumes of the *Medicago* genus have a symbiotic relationship with the bacterium Sinorhizobium meliloti and develop root nodules housing large numbers of intracellular symbionts. Members of the nodule-specific cysteine-rich peptide (NCR) family induce the endosymbionts into a terminal differentiated state. Individual cationic NCRs are antimicrobial peptides that have the capacity to kill the symbiont, but the nodule cell environment prevents killing. Moreover, the bacterial broad-specificity peptide uptake transporter BacA and exopolysaccharides contribute to protect the endosymbionts against the toxic activity of NCRs. Here, we show that other S. meliloti functions participate in the protection of the endosymbionts; these include an additional broad-specificity peptide uptake transporter encoded by the *yejABEF* genes and lipopolysaccharide modifications mediated by *lpsB* and *lpxXL*, as well as *rpoH1*, encoding a stress sigma factor. Strains with mutations in these genes show a strain-specific increased sensitivity profile against a panel of NCRs and form nodules in which bacteroid differentiation is affected. The *lpsB* mutant nodule bacteria do not differentiate, the *lpxXL* and *rpoH1* mutants form some seemingly fully differentiated bacteroids, although most of the nodule bacteria are undifferentiated, while the *yejABEF* mutants form hypertrophied but nitrogen-fixing bacteroids. The nodule bacteria of all the mutants have a strongly enhanced membrane permeability, which is dependent on the transport of NCRs to the endosymbionts. Our results suggest that S. meliloti relies on a suite of functions, including peptide transporters, the bacterial envelope structures, and stress response regulators, to resist the aggressive assault of NCR peptides in the nodule cells.

## INTRODUCTION

Antimicrobial peptides (AMPs) are essential mediators of innate immunity in eukaryotes. Their function is to attack and kill harmful invading microbes (1–3). Many organisms have also recruited AMPs as essential regulators of bacteria in symbiotic associations (2). In symbiosis, hosts intentionally maintain bacterial partners, and the role of “symbiotic” AMPs is therefore not to eradicate the symbiotic microbes but rather to police them or to optimize their metabolic integration with the hosts (2, 4). An extreme case of deployment of AMPs for controlling endosymbiont populations, involving hundreds of peptides, has been described for the rhizobium-legume symbiosis (2, 5–9). Legumes form a symbiosis with phylogenetically diverse nitrogen-fixing soil bacteria, collectively called rhizobia. This nutritional symbiosis provides reduced nitrogen to the plants. The symbiosis implies the formation of nodules, specific symbiotic organs, on the roots of the plants. These nodules house the nitrogen-fixing rhizobia, which transfer their produced ammonia to the plant in return for the exclusive niche in the nodules, where they multiply massively from a single bacterium or very few infecting bacteria to a population of millions.

After endocytic uptake by the symbiotic nodule cells, the multiplied bacteria reside intracellularly in vesicles called symbiosomes. The nodule cells and symbiosomes establish the optimal conditions for nitrogen fixation and metabolic exchange with the endosymbionts and, at the same time, keep them in check. The low oxygen levels prevailing in the symbiotic nodule cells transform the rhizobia into a differentiated physiological state, called the bacteroid, which is adapted for nitrogen fixation. Moreover, in certain legume clades, like the Inverted Repeat-Lacking Clade (IRLC) and the Dalbergioids, the physiological transition of the bacteroids is accompanied by a remarkable differentiation process that is manifested in an irreversible loss of the capacity of bacteroids to divide (10, 11). These terminally differentiated bacteroids have a partially permeabilized cell membrane. They are giant bacterial cells. A switch in the bacterial cell cycle, from a regular succession of replication and division to a series of repeated genome replications without divisions, drives this cell enlargement, resulting in polyploid bacteroids.

The terminal differentiation is triggered by a family of nodule-specific cysteine-rich peptides (NCRs), produced by the symbiotic nodule cells (11–13). Over 600 *NCR* genes were identified in the Medicago truncatula genome, while the *NCR* repertoire in other species of the IRLC and the Dalbergioids ranges from a few to several hundred (11, 12, 14). The *NCR* genes in *M. truncatula* are specifically expressed in the symbiotic cells (15). They are activated in waves during the differentiation of the bacteroids, including sets of *NCR* genes activated at the onset and others at the intermediate or final stages of the differentiation. These temporal profiles indicate that the *NCR* genes have specific functions during the bacteroid formation process.

The NCR peptides have structural features shared with AMPs, and at least some NCRs, in particular, the cationic ones, can kill or inhibit *in vitro* the growth of not only the rhizobium symbionts but also many other bacteria and even fungi (16). Their major antibacterial activity results from their capacity to disturb the integrity of the inner and outer membranes of bacteria (13, 17), although some NCRs also have activities inhibiting essential intracellular machineries (18). However, bacteroids remain metabolically active for a very long time, despite the high burden of NCRs. Possibly, the environment of the symbiotic nodule cells and symbiosomes contributes to tempering the antimicrobial activities of the peptides. Importantly, also specific functions of the bacteria themselves are NCR resistance determinants in the bacteroids.

Sinorhizobium meliloti, the symbiont of *Medicago* plants, requires the peptide transporter BacA to counter the NCR peptides inside the symbiotic nodule cells and establish a chronic infection (19). S. meliloti *bacA* mutants are hypersensitive to the antimicrobial NCRs. They induce nodules and infect plant cells in a seemingly normal fashion, but the mutants die rapidly after their release in the symbiotic cells. This death can be avoided by blocking NCR transport to the infecting rhizobia in the *M. truncatula dnf1* mutant (19). BacA proteins are broad-specificity peptide uptake transporters (20–22). They can promote the uptake of NCR peptides, suggesting that BacA provides resistance by redirecting the peptides away from the bacterial membrane, thereby limiting membrane damage. Exopolysaccharide (EPS) is another known factor of S. meliloti that helps the endosymbionts to withstand the NCRs (14, 23). This negatively charged extracellular polysaccharide traps the cationic AMPs, reducing their effective concentration in the membrane vicinity.

Bacterial resistance to AMPs is usually multifactorial (24), suggesting that besides BacA and EPS, additional functions of S. meliloti bacteroids contribute to resisting the NCRs in the symbiotic nodule cells. The literature on S. meliloti is rich in the description of bacterial genes that are required for symbiosis. However, the reporting on these mutants often lacks precise information on their bacteroid phenotype and/or on their sensitivity to NCRs. Moreover, transcriptome sequencing (RNA-seq) and transposon sequencing (Tn-seq) analyses of NCR-treated cells and NCR-protein interaction studies identified a whole suite of additional candidate NCR-responsive functions in S. meliloti (18, 25–27). Together with BacA and EPS, some of these S. meliloti functions may contribute to alleviate the NCR stress on the bacteroids. To test this hypothesis, we have selected candidate genes and analyzed the phenotype of the corresponding mutants in NCR resistance and bacteroid formation.

## RESULTS

### Sinorhizobium meliloti mutants with enhanced sensitivity to NCR peptides.

The S. meliloti functions selected in this study include a broad-specificity peptide uptake transporter encoded by the *yejABEF* genes (SMc02829 to SMc02832) and lipopolysaccharide (LPS) modifications mediated by *lpsB* (SMc01219) and *lpxXL* (SMc04268), as well as *rpoH1* (SMc00646), encoding a stress sigma factor. The YejABEF ABC transporter was selected on the basis of a genetic screen by Tn-seq of S. meliloti, revealing that mutants have an increased sensitivity to the peptide NCR247 (25). Moreover, another Tn-seq study revealed that the transporter is essential for symbiosis (28). The LPS structure is one of the major determinants of AMP resistance and sensitivity in Gram-negative bacteria (24, 29). The selected genes *lpsB* and *lpxXL* encode a glycosyltransferase involved in the synthesis of the LPS core and a very-long-chain fatty acid acyltransferase involved in the biosynthesis of lipid A, respectively. Strains with mutations in these genes are affected in their resistance to AMPs and in symbiosis (30, 31). Finally, the *rpoH1* gene is a global stress response regulator in S. meliloti (32). This gene, as well as its target genes, is upregulated in NCR247-treated cells (26, 27). An *rpoH1* mutant is also affected in symbiosis (33). A second gene, *rpoH2*, has no apparent function under free-living conditions or during symbiosis (34, 35). These selected genes are expressed in all nodule zones but have peak expression in different regions of the nodule, where bacteria infect plant cells, undergo the differentiation process, or fix nitrogen (36) (see Fig. S1 in the supplemental material).

10.1128/mBio.00895-21.1 FIG S1 Expression pattern in nodules of bacterial and *NCR* genes. (A) Schematic drawing of a *Medicago* nodule organized in functional zones. The meristem is bacterium free and contains dividing cells, allowing the organ to grow. In the early infection zone, bacteria proliferate in infection threads (green lines) and are released by endocytosis inside cells derived from the meristem. In the late infection zone and the so-called interzone, the bacteria differentiate into bacteroids. The fixation zone contains the fully differentiated, nitrogen-fixing bacteroids. The pictures show bacteria inside the nodule cells and are presented at the same scale (bar = 1 μm), allowing one to appreciate the transformation of the bacteria. (B) Against the backdrop of a changing landscape of NCR peptides (rainbow colors schematically represent peptides appearing and disappearing at different stages of symbiotic cell differentiation), the bacterial functions described in this study are critical at distinct stages of the bacteroid differentiation process. It should be noted that the functions that are essential in early stages, such as LpsB and BacA, can also be important in later stages of the bacteroid differentiation. However, the phenotypic analysis of the corresponding mutants cannot reveal these putative late roles. (C, D) The relative expression profiles (percentages of the total) of the studied bacterial genes *lpsB*, *lpxXL*, *rpoH1*, *yejF*, *yejE*, *yejB*, *yejA*, and *bacA* (C) and of *NCR280*, *NCR247*, *NCR183*, and *NCR169* (D) in the meristem, early infection zone, late infection zone, interzone, and fixation zone of *M. truncatula* nodules are displayed. The expression patterns of the S. meliloti *nifH* gene, encoding a nitrogenase subunit, and of the *M. truncatula* leghemoglobin genes (*MtLb*) are included in panels C and D, respectively, as a reference indicating the completion of bacteroid formation and the onset of nitrogen fixation. Data were extracted from Guefrachi et al. (BMC Genomics 15:712, https://doi.org/10.1186/1471-2164-15-712) and were obtained by transcriptome sequencing (RNA-seq) analysis of laser-microdissected nodule tissues (Roux et al., Plant J 77:817–837, https://doi.org/10.1111/tpj.12442). Download FIG S1, PDF file, 0.2 MB. Copyright © 2021 Nicoud et al. 2021 Nicoud et al. https://creativecommons.org/licenses/by/4.0/ This content is distributed under the terms of the Creative Commons Attribution 4.0 International license.

**FIG 1:**
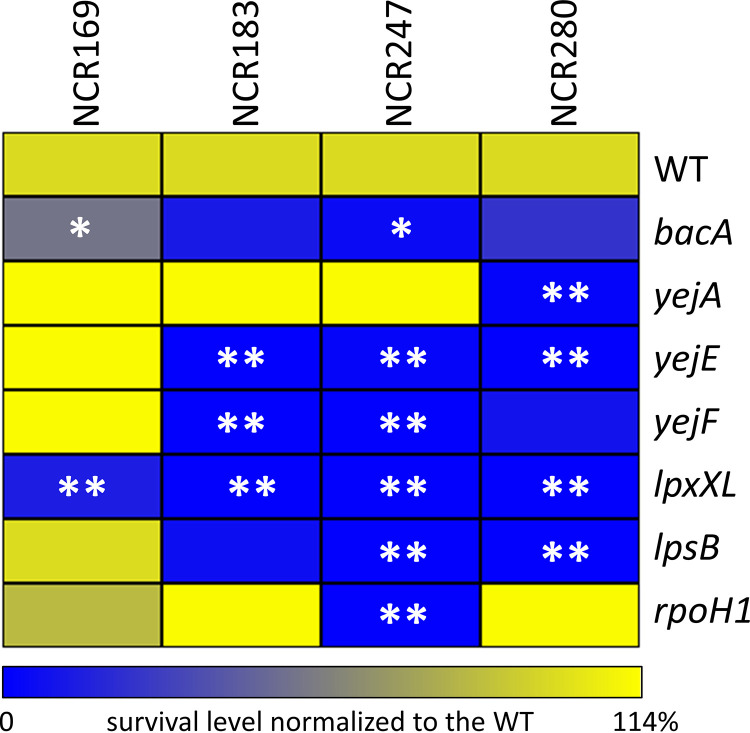
Sensitivity profile of Sinorhizobium meliloti strains to a panel of NCR peptides. The heatmap shows the survival of the mutant strains, expressed as percentages of that of the wild type (WT), set at 100%, for each peptide treatment. Nonparametric Kruskal-Wallis and *post hoc* Dunn tests were used to assess the significance of differences, which is indicated by asterisks (**, *P < *0.05; *, *P < *0.1). The results of one representative experiment out of two are shown.

The sensitivities of strains with mutations in these candidate genes (Table S1) were tested with a small panel of cationic NCR peptides that were previously shown to have antimicrobial activities (14). The tested peptides, NCR169 (isoelectric point, 8.45), NCR183 (10.10), NCR247 (10.15), and NCR280 (9.80), displayed three different expression patterns in the nodule tissues (15). The *NCR280* gene is expressed in the younger nodule cells and the *NCR169* gene in the older cells, while *NCR183* and *NCR247* have an intermediate expression pattern (Fig. S1). The selected mutants were tested along with the wild-type strain and the *bacA* mutant, which was previously shown to be hypersensitive to NCRs (19). The four tested NCR peptides had strong antimicrobial activities against the wild-type strain, which displayed a survival rate ranging from 8% to 0.03%, depending on the tested peptide (Table S2). In agreement with previous results, the *bacA* mutant was hypersensitive to the four peptides (Table S2; Fig. 1). Interestingly, the newly analyzed mutants all displayed a higher sensitivity to at least one of the peptides than the wild type (Table S2; Fig. 1). The *lpxXL* mutant was more sensitive to the four peptides, and the *lpsB*, *yejE*, and *yejF* mutants were more sensitive to NCR183, NCR247, and NCR280. The *yejA* mutant was more sensitive to NCR280, and the *rpoH1* mutant was more sensitive to NCR247. The differential responses to peptide NCR247 of the *yejA* mutant, on one hand, and of the *yejE* and *yejF* mutants, on the other hand, correspond to results of the previously described Tn-seq screen with this peptide (25). In contrast to the other mutants, the strains with mutations in the YejABEF transporter genes were newly constructed and not characterized before. Therefore, we confirmed that the NCR247 sensitivity phenotype was directly attributable to the inactivation of the transporter by complementation of the *yejF* mutant phenotype with a plasmid-borne copy of the *yejABEF* genes (Table S2). Taken together, our analysis indicated that each mutant displayed a sensitivity profile specific to the panel of tested peptides.

10.1128/mBio.00895-21.9 TABLE S1 Strains and primers used for mutant constructions. Download Table S1, PDF file, 0.2 MB. Copyright © 2021 Nicoud et al. 2021 Nicoud et al. https://creativecommons.org/licenses/by/4.0/ This content is distributed under the terms of the Creative Commons Attribution 4.0 International license.

10.1128/mBio.00895-21.10 TABLE S2 Survival after NCR treatment of wild-type and mutant Sinorhizobium meliloti strains. Download Table S2, PDF file, 0.6 MB. Copyright © 2021 Nicoud et al. 2021 Nicoud et al. https://creativecommons.org/licenses/by/4.0/ This content is distributed under the terms of the Creative Commons Attribution 4.0 International license.

Inside the nodules, bacteroids experience (besides exposure to the NCR peptides) additional stress factors, such as elevated hydrogen peroxide levels (37), an acidic pH formed in the peribacteroid space (38), and a microaerobic environment (39). Almost all the mutants behaved very similarly to the wild type during unstressed growth in culture or in response to hydrogen peroxide, acid, and low-oxygen stress (Fig. S2). Only the *rpoH1* mutant was much less resistant to acid, in agreement with previous reports (33, 40), as well as to anaerobic stress, and it had a reduced growth rate (Fig. S2). On the other hand, the mutants were more sensitive than the wild type to the exposure to the detergent sodium dodecyl sulfate (SDS) (Fig. S2), indicating that these mutants have a reduced ability to cope with membrane-permeabilizing stresses, in agreement with their increased NCR sensitivity. Thus, except for the *rpoH1* mutant, which has a general defect in growth and stress management, the selected mutants seem to be, among the known stress factors in the nodule environment, specifically sensitive to the NCRs.

10.1128/mBio.00895-21.2 FIG S2 Phenotypes of Sinorhizobium meliloti mutants under *in vitro* stress conditions. (A) Growth inhibition in the presence of H_2_O_2_ (*n* = 10); (B) growth inhibition in the presence of HCl (*n* = 10); (C) growth inhibition under microoxia (*n* = 4); (D) growth inhibition under anoxia (*n* = 4); (E) growth curves and growth rate in liquid medium (*n* = 10); (F) growth inhibition in the presence of SDS (*n* = 10). Growth inhibition was determined by the size of a halo formed around a paper patch soaked in the stress compound (A, B, F) and by colony forming unit (CFU) counting (C, D). Error bars in all panels are standard deviations. Nonparametric Kruskal-Wallis and *post hoc* Dunn tests were used to assess the significance of differences in the data of panels A to F (*, *P < *0.05; **, *P < *0.01; ***, *P < *0.001). The results of one representative experiment out of at least two are shown. Download FIG S2, PDF file, 0.8 MB. Copyright © 2021 Nicoud et al. 2021 Nicoud et al. https://creativecommons.org/licenses/by/4.0/ This content is distributed under the terms of the Creative Commons Attribution 4.0 International license.

### Nodule formation by NCR-sensitive Sinorhizobium meliloti mutants.

Next, the phenotypes of these mutants in symbiosis with *M. truncatula* were compared with those of the wild-type strain and the *bacA* mutant. Measurement of the nitrogen fixation activity and macroscopic inspection of the root systems of plants inoculated with the wild type and the seven mutants (Fig. S3) revealed that, besides the wild type, strains with mutant *yejA*, *yejE*, *yejF*, or *lpxXL* genes formed functional nodules (Fix^+^), although the *yejE*, *yejF*, and *lpxXL* mutants had a reduced nitrogen fixation activity. On the other hand, the *bacA*, *lpsB*, and *rpoH1* mutants formed nonfunctional (Fix^–^) and abnormal-looking nodules that were small and white, in agreement with previous descriptions (19, 30, 33).

10.1128/mBio.00895-21.3 FIG S3 Symbiotic phenotypes of Sinorhizobium meliloti NCR-sensitive mutants in Medicago truncatula. (A) Nitrogen fixation activity determined by the acetylene reduction assay on whole roots of nodulated plants infected with the indicated bacterial mutants at 21 days post inoculation. NI, noninoculated control plants; WT, plants nodulated by the wild-type strain Sm1021. Boxplots were generated from 15 plants each. Letters associated with each condition represent statistically different classes determined by a nonparametric Dunn test, with an α threshold equal to 0.05. (B) Nodule phenotypes at 21 days post inoculation. Arrowheads indicate small nodules elicited by the *lpsB* mutant. The scale bar (2 mm) applies to all panels. Download FIG S3, PDF file, 0.7 MB. Copyright © 2021 Nicoud et al. 2021 Nicoud et al. https://creativecommons.org/licenses/by/4.0/ This content is distributed under the terms of the Creative Commons Attribution 4.0 International license.

**FIG 2:**
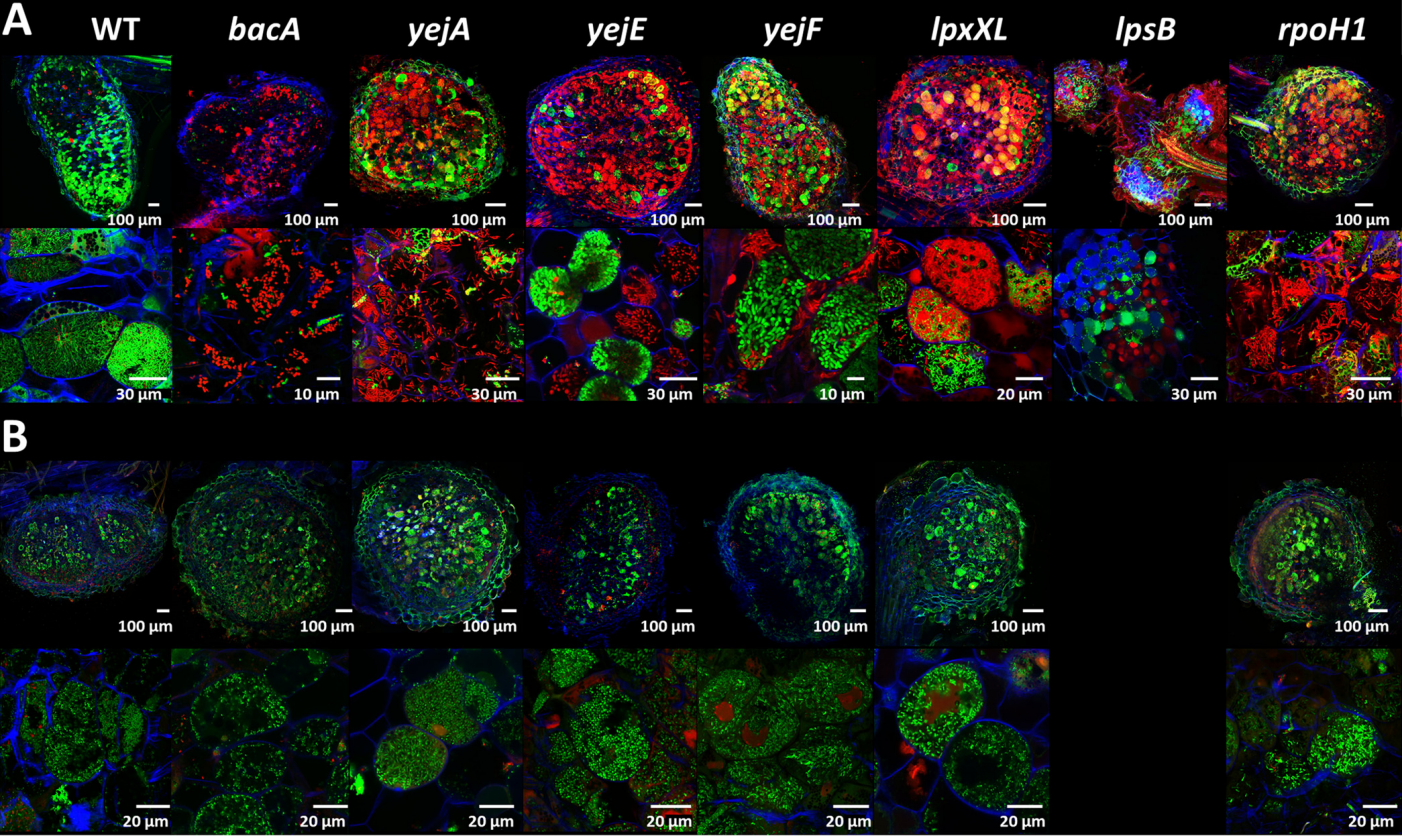
Symbiotic phenotypes of Sinorhizobium meliloti mutants during symbiosis with wild-type Medicago truncatula or the *dnf1* mutant. (A) Membrane permeability in bacteroids of the S. meliloti Sm1021 wild type and mutants in wild-type *M. truncatula* nodules, determined by live-dead staining of nodule sections and confocal microscopy. (B) Membrane permeability of the S. meliloti Sm1021 wild type and mutants in nodules of the *M. truncatula dnf1* mutant, determined by live-dead staining of nodule sections and confocal microscopy. Nodule phenotype at 21 days post inoculation. (Top row) Full nodule sections; (bottom row) enlarged images of symbiotic cells. Scale bars are indicated in each panel. The staining procedure of nodule sections with a mixture of the dyes propidium iodide, SYTO 9, and calcofluor white highlights the bacteroids and nodule bacteria with a green fluorescence signal (SYTO 9) when their membranes are well preserved and with a red fluorescence (propidium iodide) when their membranes are highly permeable. Plant cell walls are stained blue (calcofluor white). Representative images are shown, originating from at least two independent nodulation assays and from 5 to 10 nodules analyzed per condition.

The histological organization of the nodules formed by the mutants, the formation of infected symbiotic cells, and the viability of the bacteria that they contain were analyzed using confocal microscopy (Fig. 2A). The used staining procedure highlights the nodule bacteria with a green fluorescence signal (SYTO 9) when their membranes are well preserved and with a red fluorescence signal (propidium iodide) when their membranes are highly permeable. As previously reported, wild-type nodules formed symbiotic cells infected with green-labeled elongated bacteroids, while the nodules infected with the *bacA* mutant contained symbiotic cells carrying small undifferentiated bacteria stained red (19).

In contrast to what we expected from the macroscopic inspection of the nodules and their ability to fix nitrogen, the *yejA*, *yejE*, and *yejF* mutant bacteroids were substantially altered compared to wild-type bacteroids. A high proportion of them were stained red, indicating that their membranes were strongly permeabilized. Nevertheless, other host cells contained bacteroids stained green by the SYTO 9 dye. LpxXL is known to be important, but not essential, to S. meliloti during symbiosis with alfalfa (*Medicago sativa*) (41). The mutant forms hypertrophied bacteroids (31). In our experiments, the *lpxXL* mutant displayed elongated bacteroids that were mostly permeable to propidium iodide (stained red). This observation confirmed that S. meliloti LpxXL is also essential for the normal bacteroid differentiation process in M. truncatula but that it is not crucial for infection and nitrogen fixation. The *rpoH1* mutant formed elongated bacteroids that nevertheless were strongly stained by propidium iodide, in agreement with the Fix^–^ phenotype of the nodules and the described phenotype of the mutant in alfalfa (33). Inside the small bumps elicited by the *lpsB* mutant, no cell seemed to be colonized by bacteria, as revealed by confocal microscopy. Thus, the S. meliloti *lpsB* mutant failed to colonize the host cells and was defective at a stage before plant cell infection. It was previously reported that the *lpsB* mutant colonizes nodule cells in *M. sativa*, suggesting a more severe defect in *M. truncatula* (42, 43). Therefore, we tested the phenotype of all the selected mutants also on *M. sativa* (Fig. S4). The *lpsB*, *rpoH1*, and the *yejA* mutants indeed had milder defects on this host, while the other mutants had similar phenotypes on the two hosts.

10.1128/mBio.00895-21.4 FIG S4 Symbiotic phenotype of Sinorhizobium meliloti mutants during symbiosis with *Medicago sativa*. (A) Leaves of plants inoculated with the indicated bacterial strains. Scale bar = 2 cm. (B) Nodule phenotype at 21 days post inoculation. Scale bar = 1 mm. (C) Bacteroid viability determined by live-dead staining of nodule sections and confocal microscopy. (Top row) Full nodule sections; (bottom row) enlarged images of symbiotic cells. Scale bars are indicated in each panel. (D) Composite image of a *yejF* mutant-infected nodule section showing the rapid permeabilization of the membranes of the internalized bacteria (red staining). Note that the bacteria in infection threads (white arrows) are not permeabilized (green staining). The images of the composition are separated by dashed lines. (E) Nitrogen fixation activity determined by the acetylene reduction assay on whole roots of nodulated plants infected with the indicated bacterial mutants at 21 days post inoculation. NI, noninoculated control plants; WT, plants nodulated by the wild-type strain Sm1021. Boxplots were generated from 15 plants each. Letters associated with each condition represent statistically different classes determined by a nonparametric Dunn test, with an α threshold equal to 0.05. Download FIG S4, PDF file, 1.0 MB. Copyright © 2021 Nicoud et al. 2021 Nicoud et al. https://creativecommons.org/licenses/by/4.0/ This content is distributed under the terms of the Creative Commons Attribution 4.0 International license.

### Can the increased membrane permeability of the NCR-sensitive Sinorhizobium meliloti mutants be attributed to the NCRs in nodules?

The high membrane permeability of the *bacA* mutant nodule bacteria is the result of the action of the NCR peptides in the nodule cells of this NCR-hypersensitive mutant (19). Possibly, the same is true for the other mutants. To test this hypothesis, we made use of the *M. truncatula dnf1* mutant, which is defective in a nodule-specific subunit of the signal peptidase complex (44). This mutant cannot transport NCR peptides to the symbiosomes (13). As a result, the wild-type S. meliloti nodule bacteria in this mutant are not differentiated. The *bacA* mutant, which is strongly permeabilized and stained by propidium iodide in nodules of wild-type *M. truncatula* plants, is not so in the *dnf1* mutant nodule cells because the bacteria are not challenged anymore with the NCRs (Fig. 2B) (19). Similarly to the *bacA* mutant, the *yejA*, *yejE*, *yejF*, *lpxXL*, and *rpoH1* mutants did not display membrane permeability, as revealed by the absence of propidium iodide staining, in the infected nodule cells of the *dnf1* mutant (Fig. 2B). This thus suggests that these mutants become membrane permeabilized by the actions of the NCRs and that their symbiotic defects are at least in part due to their hypersensitivity to the NCRs. The *lpsB* mutant did not form detectable nodule-like structures on the *dnf1* roots. Therefore, we cannot draw conclusions about the involvement of the NCR peptides in the symbiotic phenotype of this mutant.

### Bacteroid differentiation of the NCR-sensitive Sinorhizobium meliloti mutants.

**FIG 3:**
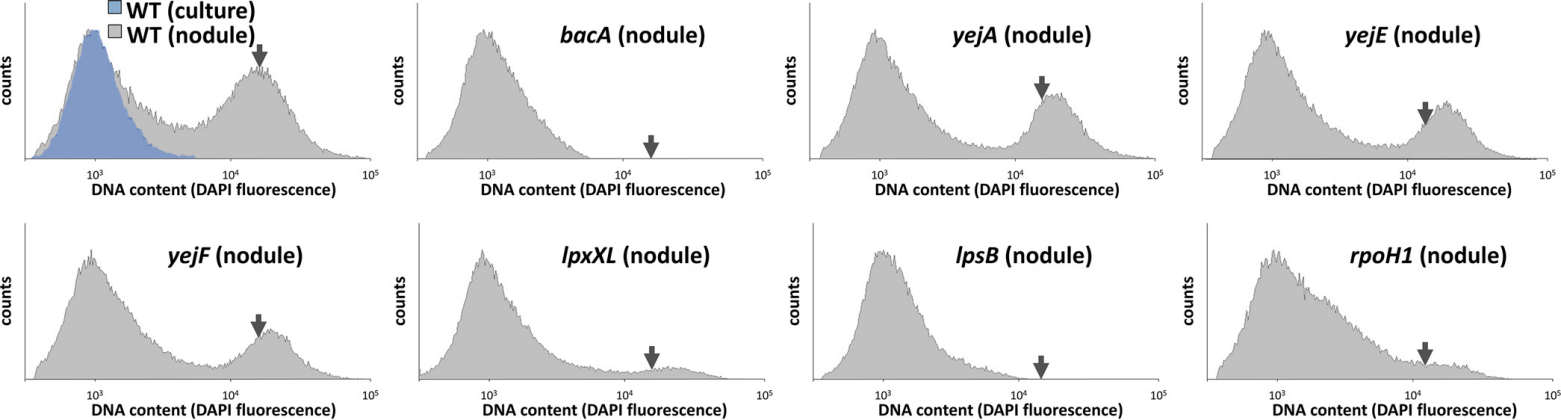
DNA contents of nodule bacteria in Medicago truncatula. Flow cytometry analysis of the DNA contents of bacteria in culture or isolated from nodules infected with the indicated strains and stained with 4′,6-diamidino-2-phenylindole (DAPI). The cell counts (*y* axes) are represented as a function of the DAPI fluorescence intensity (*x* axes). The arrow in each graph indicates the mean DNA content of wild-type bacteroids, as in the upper left panel. The results of one representative experiment out of two are shown.

A very strong cell enlargement and an increase in the ploidy level of the bacteria characterize the differentiated bacteroids in *Medicago* nodules. These parameters can readily be measured by DAPI (4′,6-diamidino-2-phenylindole) staining and flow cytometry (10). The wild-type bacteroids from *M. truncatula* nodules had a high DNA content, over 20-fold higher than the DNA content in free-living S. meliloti (Fig. 3), and increased light-scattering parameters reflecting the cell enlargement (Fig. S5). As previously reported, the nodules infected by the *bacA* mutant did not contain differentiated bacteria (Fig. 3; Fig. S5) (21). The bacteria in nodules induced by the *lpsB* mutant, probably located in infection threads, had a profile that confirmed the complete absence of differentiated bacteria (Fig. 3; Fig. S5). Nodules infected with the *rpoH1* mutant had mostly undifferentiated bacteria, although a small amount of fully differentiated cells was detected, as were cells at an intermediate stage. Also, the nodules of the *lpxXL* mutant contained many undifferentiated bacteria as well as fully differentiated ones (Fig. 3; Fig. S5). In contrast, the *yejA*, *yejE*, and *yejF* mutant nodules contained large numbers of fully differentiated bacteria (Fig. 3; Fig. S5).

10.1128/mBio.00895-21.5 FIG S5 Morphological parameters in nodule bacteria of Medicago truncatula nodules. Flow cytometry analysis of the morphology of bacteria isolated from nodules infected with the indicated strains. The forward scatter (FSC; *y* axes) is represented as a function of the side scatter (SSC; *x* axes). An “X” indicates the peak value for each sample. The positions of the peak values in the cultured bacteria and in the wild-type bacteroids are indicated in each panel by the hatched lines. Download FIG S5, PDF file, 0.8 MB. Copyright © 2021 Nicoud et al. 2021 Nicoud et al. https://creativecommons.org/licenses/by/4.0/ This content is distributed under the terms of the Creative Commons Attribution 4.0 International license.

It was reported previously that the *lpxXL* mutant formed nodules containing hypertrophied and larger bacteria than the wild-type bacteroids (31). In our study, this difference was detectable in the flow cytometry measurements, which showed a higher DAPI fluorescence and light scattering for the small portion of differentiated bacteria in these nodules (Fig. 3; Fig. S5). Moreover, we noticed that the bacteroids in the nodules infected with the *yejA*, *yejE*, and *yejF* mutants displayed similar higher levels of DNA fluorescence and light scattering (Fig. 3; Fig. S5), suggesting that these bacteroids also have abnormal morphologies.

These patterns in *M. truncatula* nodules were overall similar to those in *M. sativa*, although the *rpoH1* mutant showed a higher number of intermediate and fully differentiated bacteroids and the *yejA* mutant had a profile similar to that in nodules of the wild type at 21 days post inoculation (dpi), while larger-than-normal bacteroids were detected only at 32 dpi (Fig. S6). These differences corresponded well with the macroscopic and microscopic differences in the nodules between the two host plants (Fig. 2; Fig. S3 and S4).

10.1128/mBio.00895-21.6 FIG S6 DNA content and morphological parameters in nodule bacteria in *Medicago sativa*. (A) Flow cytometry analysis of the DNA contents of bacteria in culture or isolated from nodules infected with the indicated strains and stained with 4′,6-diamidino-2-phenylindole (DAPI). The cell counts (*y* axes) are represented as a function of the DAPI fluorescence (*x* axes). The arrow in each panel indicates the mean DNA content of wild-type bacteroids. The rows represent three sets of independent experiments, and the values of DAPI fluorescence cannot be compared between experiments because of differences in instrument settings. The first two rows are samples prepared from 21 dpi nodules; the third row is from 32 dpi nodules. The latter shows that the phenotype of the *yejA* mutant evolves in older nodules. (B) Flow cytometry analysis of the morphology of bacteria isolated from nodules infected with the indicated strains. The forward scatter (*y* axes) is represented as a function of the side scatter (*x* axes). Download FIG S6, PDF file, 1.1 MB. Copyright © 2021 Nicoud et al. 2021 Nicoud et al. https://creativecommons.org/licenses/by/4.0/ This content is distributed under the terms of the Creative Commons Attribution 4.0 International license.

### Defective bacteroid differentiation of *yejE* and *yejF* mutants.

**FIG 4:**
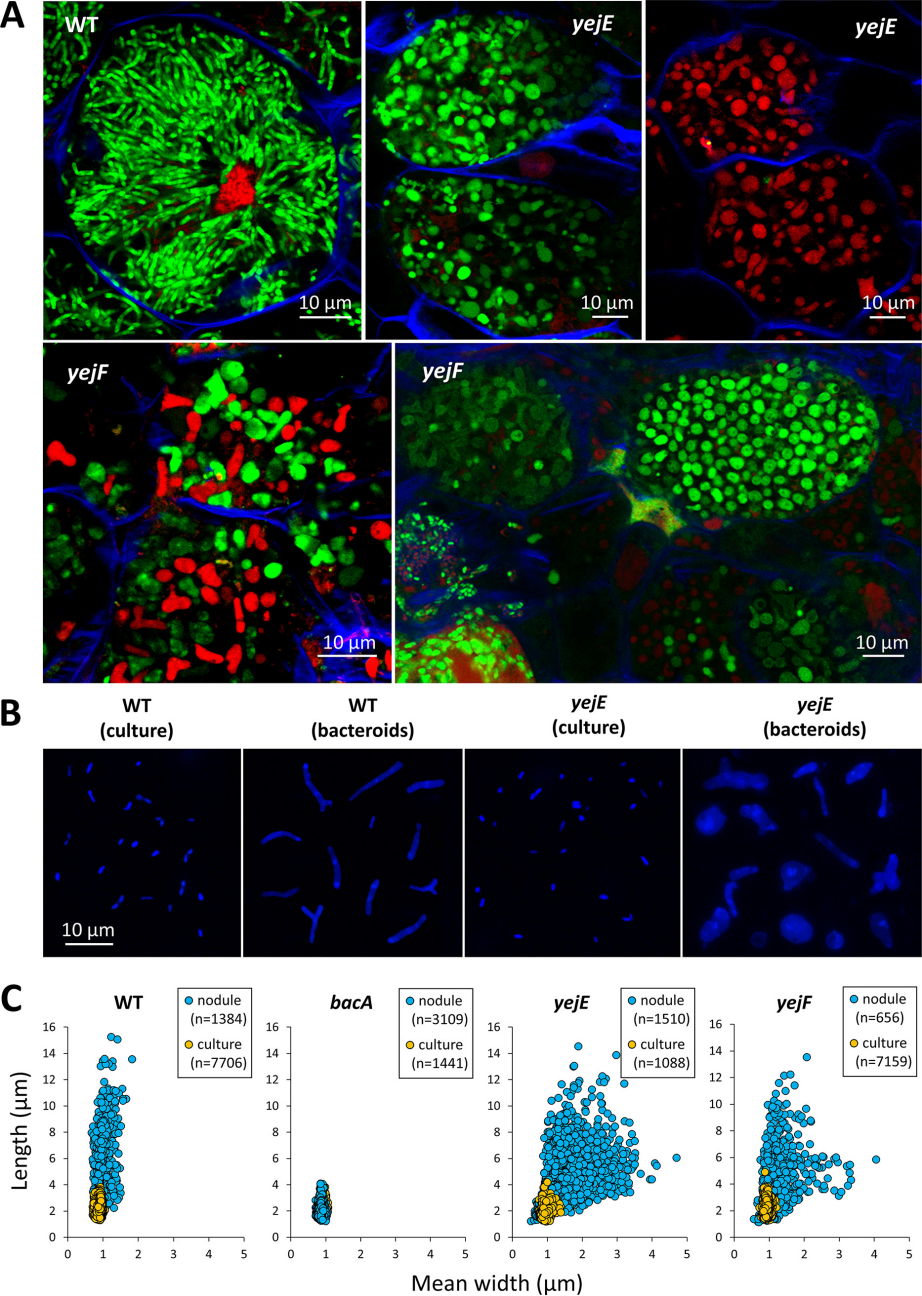
Bacteroid morphology of *yejE* and *yejF* mutants in *Medicago sativa* nodules. (A) Sections of nodules infected with the wild type and the *yejE* or *yejF* mutant were stained with a mixture of the dyes propidium iodide, SYTO 9, and calcofluor white and observed by confocal microscopy. Scale bars (10 μm) are indicated in each panel. Representative images are shown, originating from at least two independent nodulation assays and from 5 to 10 nodules analyzed per condition. (B) Preparations of cultured bacteria or purified *M. sativa* nodule bacteria of the wild type and the *yejF* mutant were observed by fluorescence microscopy. The panels are composite images, and the shown individual cells were cut from original images and recombined in a single panel. Each panel is at the same magnification, and the scale bar (10 μm) is indicated in the left panel. (C) Cell shape parameters of free-living bacteria and bacteroids determined by MicrobeJ. Dot plots show the mean widths and lengths of cells of the indicated strains. Each point represents the values of one bacterium. Numbers of analyzed cells (n) are indicated.

To confirm the altered bacteroid morphologies of the *yej* mutants suggested by the cytometry analysis, the *yejE* and *yejF* mutants were observed at high magnification by microscopy. Confocal and transmission electron microscopy (TEM) of *M. sativa* nodule sections showed that the nodule cells infected with the *yejE* or *yejF* mutant contained a very heterogeneous population of abnormal bacteroid morphs, including elongated, spherical, club-shaped, or irregular blob-like cells (Fig. 4A; Fig. S7). These cells contrasted strongly with the narrow, elongated wild-type bacteroids. This aberrant bacteroid phenotype of the mutants was restored to a wild-type phenotype by the introduction of a plasmid-borne copy of the *yejABEF* genes (Fig. S7; Table S1). The difference between wild-type and mutant bacteroids was also obvious by fluorescence microscopy observations of purified nodule bacteria (Fig. 4B). The quantification of cell morphology parameters by image analysis with MicrobeJ of free-living bacteria and the purified nodule bacteria confirmed the irregular forms of *yejE* and *yejF* bacteroids, notably showing that these bacteroids are broader and more spherical than the elongated wild-type bacteroids (Fig. 4C). Transmission electron microscopy further showed that the cytoplasm and inner membranes of many of the *yejF* bacteroids were retracted, leaving very large intermembrane spaces, which in some cases even developed into vacuoles entirely surrounded by cytoplasm. Some cells had multiple small and large vacuoles (Fig. S7A). Unlike with the bacteroids, in the cultured bacteria, no differences were observed in the ultrastructure of the wild-type and *yejF* mutant bacteria (Fig. S7B).

10.1128/mBio.00895-21.7 FIG S7 Cellular defects in the *yejF* mutant bacteroids in *Medicago sativa* nodules. (A) Transmission electron microscopy of wild-type (WT) and *yejF* mutant bacteroids. The arrows indicate retracted inner membranes in the *yejF* bacteroids; “v” indicates vacuoles, and “*” indicates an enlarged peribacteroid space found in the angle of a branched bacteroid. (B) Ultrastructure of wild-type and *yejF* mutant bacteria in culture by transmission electron microscopy. Scale bars = 1 μm. (C) Confocal microscopy of sections of nodules infected with wild-type, *yejE* mutant, or *yejF* mutant bacteria carrying the empty plasmid pSRK-Gm or the complementing plasmid pSRK-*yejABEF*. Download FIG S7, PDF file, 0.8 MB. Copyright © 2021 Nicoud et al. 2021 Nicoud et al. https://creativecommons.org/licenses/by/4.0/ This content is distributed under the terms of the Creative Commons Attribution 4.0 International license.

**FIG 5:**
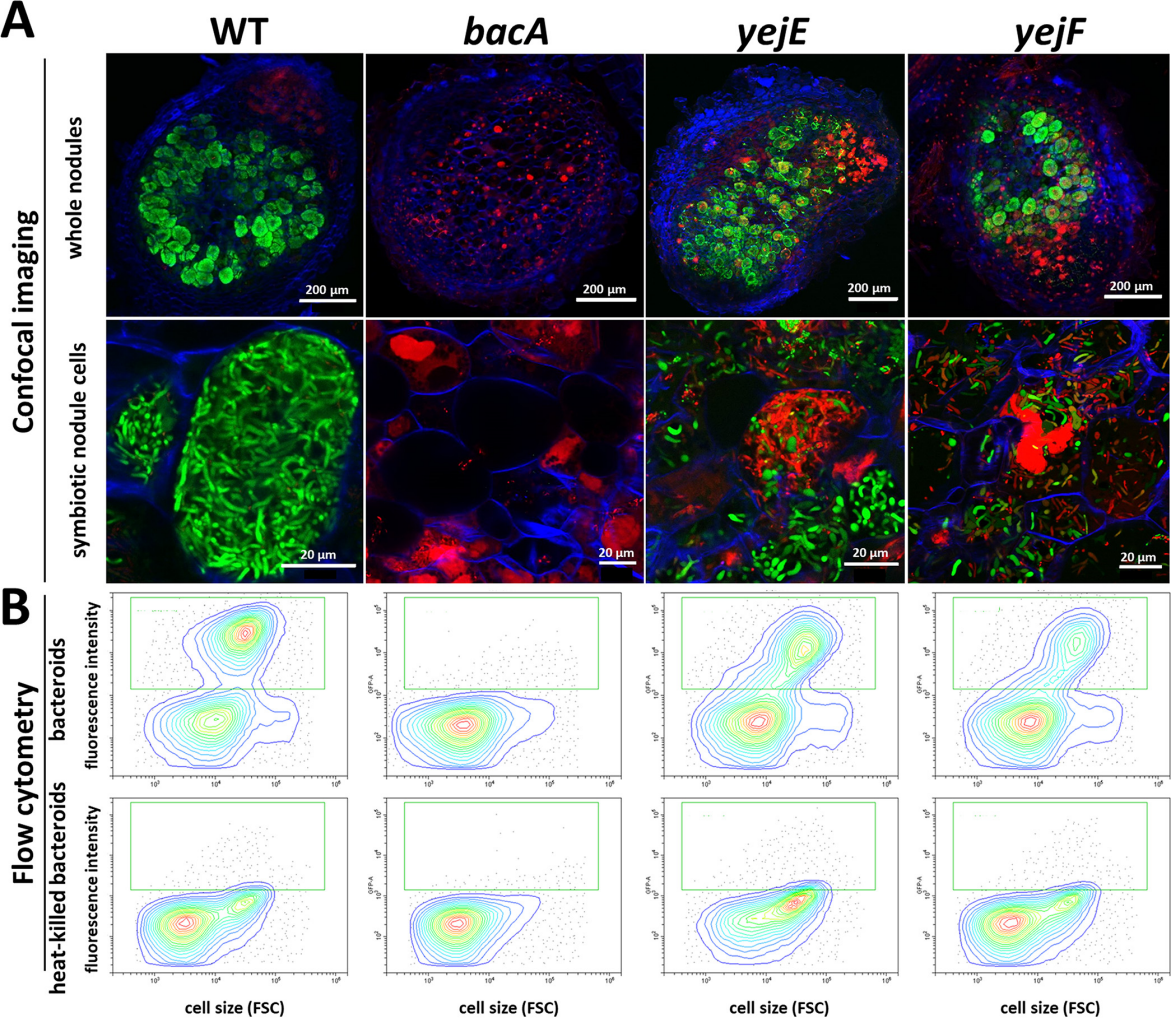
Nitrogenase expression in the *yejE* and *yejF* mutant bacteroids in Medicago truncatula nodules. (A) Confocal microscopy of sections of nodules infected with S. meliloti Sm1021.pHC60-p*nifH*::*GFP* (WT), Sm1021.Δ*bacA*.pHC60-p*nifH*::*GFP* (*bacA*), Sm1021.Δ*yejE*.pHC60-p*nifH*::*GFP* (*yejE*), or Sm1021.Δ*yejF*.pHC60-p*nifH*::*GFP* (*yejF*) and stained with propidium iodide (red stain). Green-stained bacteroids are functional, while red-stained bacteroids are nonfunctional. (B) Flow cytometry determination of GFP levels in nodule bacteria (upper panels) and heat-killed nodule bacteria (lower panels). The green square shows the position of the GFP-positive bacteroids. FSC, forward scatter.

The formation of strongly abnormal bacteroids of the *yej* mutants is *a priori* contradictory with the nitrogen fixation activity of these nodules. Expression of the *nifH* gene is a marker for nitrogen-fixing bacteroids (45). A green fluorescent protein (GFP) gene under the control of the *nifH* promoter was introduced into the wild-type strain and the *bacA*, *yejE*, and *yejF* mutants (Table S1). Analysis of the GFP fluorescence in nodule bacteria of *M. truncatula* (Fig. 5) and *M. sativa* (Fig. S8) by confocal microscopy and flow cytometry showed that despite their aberrant morphology, the *yejE* and *yejF* bacteroids were mostly functional, although some nodule cells contained highly permeable bacteroids lacking the GFP signal, suggesting that they were nonfunctional.

10.1128/mBio.00895-21.8 FIG S8 Nitrogenase expression in the *yejE* and *yejF* mutant bacteroids in *Medicago sativa* nodules. (A) Confocal microscopy of sections of nodules infected with S. meliloti Sm1021.pHC60-p*nifH*::*GFP* (WT), Sm1021.Δ*bacA*.pHC60-p*nifH*::*GFP* (*bacA*), Sm1021.Δ*yejE*.pHC60-p*nifH*::*GFP* (*yejE*), or Sm1021.Δ*yejF*.pHC60-p*nifH*::*GFP* (*yejF*) and stained with propidium iodide (red stain). Green-stained bacteroids are functional, while red-stained bacteroids are nonfunctional. (B) Flow cytometry determination of GFP levels in nodule bacteria (upper panels) and heat-killed nodule bacteria (lower panels). The green square shows the position of the GFP-positive bacteroids. Download FIG S8, PDF file, 1 MB. Copyright © 2021 Nicoud et al. 2021 Nicoud et al. https://creativecommons.org/licenses/by/4.0/ This content is distributed under the terms of the Creative Commons Attribution 4.0 International license.

### NCR247 uptake by the YejABEF transporter.

**FIG 6:**
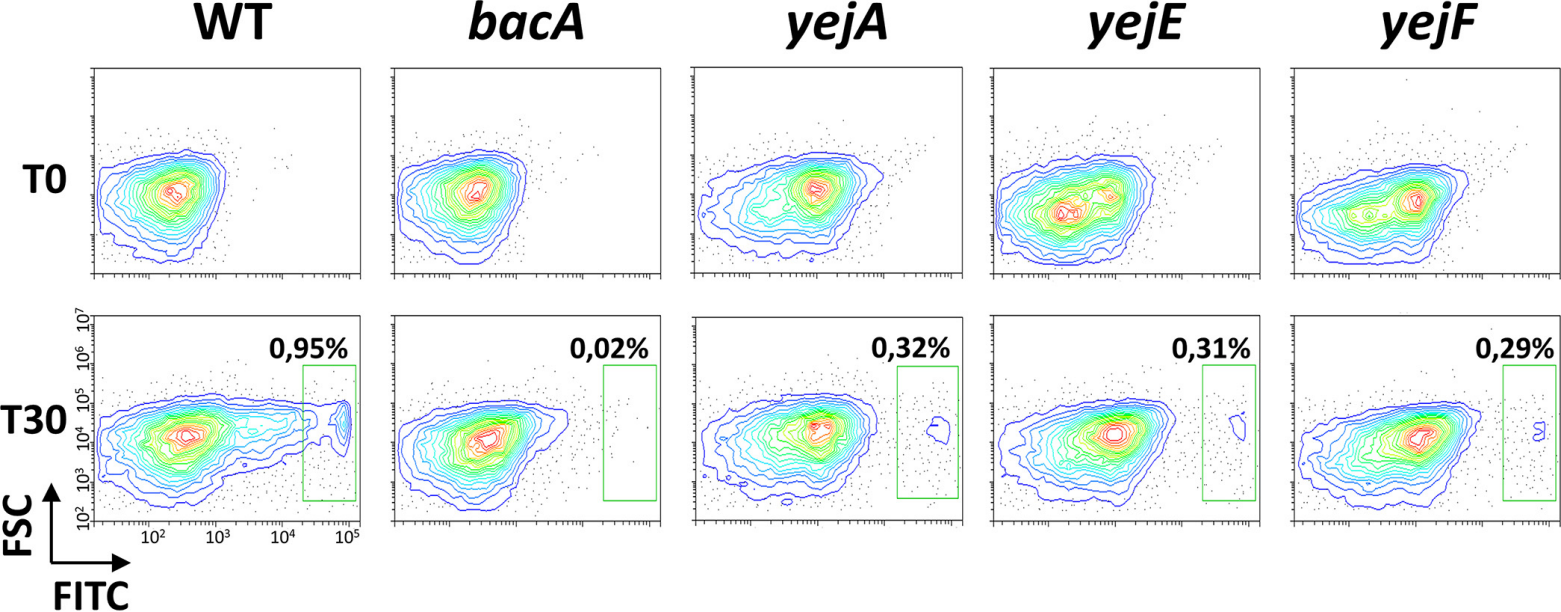
Uptake of NCR247 mediated by the BacA and YejABEF transporters. Flow cytometry measurement of fluorescein isothiocyanate (FITC) fluorescence in S. meliloti cells before (time zero [T0]) and after 30 min of incubation (T30) of the bacteria with the NCR247-FITC peptide. “FITC” on the *x* axis is FITC-derived fluorescence, and “FSC” on the *y* axis is forward scatter. In the wild type, two subpopulations of bacteria were observed, an FITC-negative population and an FITC-positive one, which has taken up the NCR247-FITC peptide. The green rectangle in the different panels shows the FITC-positive subpopulation in the wild type and the corresponding region in the analyzed mutants. In the *bacA* mutant, the FITC-positive subpopulation is absent, and in the *yejA*, *yejE*, and *yejF* mutants, it is strongly reduced compared to that of the wild type. The analysis was performed in quadruplicate, and the results of a representative example are shown.

In Escherichia coli, both YejABEF and the BacA homolog SbmA mediate the transport of microcin C peptide-nucleotide antibiotics. Therefore, we tested whether the overlap in the substrates of YejABEF and BacA can be extended to NCR peptides, which are known substrates of BacA (20, 21). We tested the impact of the mutations in the YejABEF transporter genes on NCR247 uptake and compared it with a mutation in *bacA*. With a flow cytometry-based assay and a fluorescent derivative of the NCR247 peptide, we found that while NCR247 uptake is completely abolished in the *bacA* mutant, as expected, its uptake is also reduced but not completely abolished in the *yejA*, *yejE*, and *yejF* mutants (Fig. 6). This suggests that the YejABEF transporter contributes to NCR uptake.

## DISCUSSION

### Multiple functions of Sinorhizobium meliloti contribute to NCR resistance and are required for bacteroid formation and persistence.

The NCR peptides are a two-edged sword. On the one hand, they maneuver the rhizobial endosymbionts into a terminally differentiated state via a multitude of activities on the bacteria, of which membrane permeabilization, cell cycle perturbation (polyploidization), and cell enlargement are the most visible ones. On the other hand, many NCRs, notably the cationic ones, have antimicrobial activities that potentially kill the endosymbionts (13, 14). Therefore, rhizobia have to defend themselves to be able to establish a chronic infection in the NCR-producing symbiotic cells of the nodules. Previous work has identified the BacA peptide transporter and the EPS barrier in the bacterial envelope as defenses of *Sinorhizobium* strains against the NCRs of their *Medicago* hosts (14, 19, 23).

Here, we defined three new functions in S. meliloti, the LPS, the sigma factor RpoH1, and the YejABEF peptide transporter, as additional determinants in bacteroids required to cope with NCR peptides. We show that mutants with the corresponding genes knocked out are more sensitive to a panel of antimicrobial NCRs and that this hypersensitivity is correlated with a strongly enhanced membrane permeability of the nodule bacteria and abnormalities in their morphology and ploidy levels. It is striking, however, that the different mutants have markedly different bacteroid phenotypes, ranging from undifferentiated to hyper-differentiated. This can be attributed to several factors. Each mutant has a specific NCR sensitivity profile when tested against a small panel of peptides, and the “NCR landscape” present in the developing symbiotic cells is continuously changing because of the expression of the *NCR* genes in different waves during symbiotic cell differentiation (15). Accordingly, the mutants may accumulate NCR-induced damage at different rates and reach the breaking point at different stages over the course of the bacteroid differentiation process (Fig. S1). Moreover, the expression patterns of the bacterial genes in the nodule zones are different, suggesting that their principal impact is realized at distinct stages of the symbiotic cell development and bacteroid differentiation.

Blocking the targeting of the NCR peptides to the nodule bacteria by the use of the *M. truncatula dnf1* mutant prevents the membrane permeabilization of the nodule bacteria in the mutants and results in the formation of very similar aberrant nodules by the wild-type and all mutant strains (except for the *lpsB* mutant [see below]). This observation places the symbiotic role of these bacterial genes, at least in part, downstream of the peptide targeting the symbiosomes and is in agreement with the important role of these genes in responding to the NCRs. It should be noted that besides the antimicrobial NCR peptides, other stress factors, such as high H_2_O_2_ levels, a low pH, and low oxygen, are present in the nodule environment (37–39). We found that, except for the *rpoH1* mutant, strains with mutations in the studied genes were not affected by these stresses differently from the wild type, making it unlikely that they are the main cause of the bacteroid phenotypes in these mutants. However, our analyses do not exclude the possibility that these genes also contribute in the bacteroids to other, less-well-characterized stress factors in the nodule environment.

The functions of S. meliloti described here and before probably picture only part of the full toolkit of this symbiont to survive the NCR challenge. Many additional S. meliloti mutants are described with symbiotic phenotypes that are suggestive of a similar contribution to NCR resistance (46–57). In addition, other genes that may be important for symbiosis were discovered in a Tn-seq screen with the peptide NCR247 (25). This screen identified here-described functions, like those of the BacA and YejABEF transporters and LPS and EPS biosynthesis, but also identified never-analyzed functions. It would be of major interest to explore the functions of these genes in relation to the NCR response of bacteroids. Note that some of these genes might have escaped identification in previous genetic screens for symbiosis mutants because mutations might provoke only subtle phenotypes with a weak overall effect on nitrogen fixation under standard laboratory conditions but with bacteroid alterations in morphology and persistence, as illustrated here with the *yejABEF* mutants.

### Response to NCR-induced stress regulated by the alternative sigma factor RpoH1.

Bacteria deal with different types of stress conditions by global transcriptional responses mediated by alternative sigma factors. Among them, the RpoE and RpoH sigma factors are known to respond to periplasmic and membrane stressors, including AMPs (58–60). Given the membrane damage provoked by NCRs, a role of the orthologous regulators of S. meliloti in bacteroid differentiation and the NCR response may *a priori* have been expected. S. meliloti has 11 RpoE-like sigma factors. Remarkably, despite the fact that some RpoEs have a considerable effect on gene transcription, all single mutants, all possible double mutants, and even a mutant lacking all 11 genes showed no detectable phenotypic difference from the wild type in symbiosis or during many tested free-living growth conditions, including growth in the presence of membrane stresses (61, 62). Thus, the 11 S. meliloti RpoEs do not have the expected role in regulating the envelope stress response. This role seems to be taken up by RpoH1 in S. meliloti, in agreement with the observation that the growth of the *rpoH1* mutant is affected in the presence of various membrane-disrupting agents (33). We propose that the here-uncovered role of RpoH1 in NCR resistance is connected to its regulation of membrane stress. In addition, RpoH1 is crucial to various cytoplasmic stresses, including heat and osmotic and acidic stress, as well as anoxia or microoxia (33–35, 40). Thus, besides providing resistance to NCRs, RpoH1 might also be crucial for handling these additional stress factors in bacteroids. As RpoH1 controls directly and indirectly the expression of several hundreds of genes, a future challenge will be to dissect this massive response into specific stress adaptations (32, 40).

### The lipopolysaccharide barrier against NCR membrane damage.

In Gram-negative bacteria, LPS constitutes the first point of attack of cationic AMPs. In a two-stage process, AMPs make the first electrostatic interactions with the negatively charged LPS, allowing the AMPs to approach the membrane lipids and subsequently to insert into the lipid bilayer, perforate it, and translocate into the periplasm (63). The chemical composition of LPS can influence the efficiency of the AMP attack, and pathogens have evolved mechanisms to recognize the presence of AMPs and to modify in response the composition of the LPS to lower the potency of the AMP attack (64).

S. meliloti *lpsB* encodes a glycosyltransferase that participates in the biosynthesis of the LPS core (65). The mutation of the gene affects both the core structure and O-antigen polymerization (42, 43). The O-antigen is thought to be a camouflage, masking the membrane and the charges in the membrane vicinity. In the *lpsB* mutant, the shorter O-antigen may be a less efficient shield against the NCRs, offering to the peptides an easier access to membrane-proximal charges resulting in the increased sensitivity of the *lpsB* mutant to the NCRs (this work) as well as to other AMPs (30, 43).

The *lpsB* mutant forms nodules on *M. sativa*, which contain infected cells, and it has a phenotype that is similar to that of the *bacA* mutant. However, the *lpsB* mutant forms uninfected nodules on *M. truncatula* roots. Therefore, it was not possible to test the implication of NCRs in this symbiotic phenotype with the use of the *dnf1* mutant, since the *DNF1* gene is expressed only in infected nodule cells (44). Since the *NCR* genes are nearly exclusively expressed in the infected symbiotic cells (15), the blockage of the mutant before the release of bacteria into nodule cells suggests that the LPS may provide protection against another stressor produced very early on in the infection process in *M. truncatula*. On the other hand, it was recently reported that some *NCR* genes are expressed in infected root hairs or Nod factor-stimulated root epidermal cells (66, 67). Thus, it is possible that the challenge with these early NCR peptides is already detrimental to the mutant, blocking any further progress in the infection process.

LpxXL of S. meliloti is a specific acyltransferase that introduces in the lipid A moiety of LPS the very-long-chain fatty acid 27-OHC28:0 (41). This LpxXL-dependent acylation is expected to make the lipid A hydrophobic, forming the biophysical basis for LpxXL-dependent NCR resistance. Indeed, it is known that the increased hydrophobicity of lipid A in bacteria increases the thickness of the outer layer of the outer membrane and reduces the membrane fluidity, which in its turn prevents or delays the insertion of diverse AMPs and subsequent membrane damage. Increasing lipid A hydrophobicity by introducing additional acyl chains is a well-known mechanism used by Salmonella to enhance its resistance against host AMPs during infection (64).

### The YejABEF peptide transporter provides resistance to membrane-damaging peptides and sensitizes bacteria to AMPs with intracellular targets.

A Tn-seq study has shown that transposon insertions in the S. meliloti *yejB* gene provoke a moderate growth impairment (68). Moreover, in a strain cured of the two symbiotic plasmids but not in the wild type, insertions in the four genes of the transporter resulted in a strong growth defect, signifying that a function redundant to YejABEF is encoded on one of the symbiotic plasmids (68). Additionally, the expression of the *yejA* gene, encoding the periplasmic binding protein of the transporter, was found in a systematic analysis of periplasmic binding proteins in S. meliloti to be induced by taurine, valine, isoleucine, and leucine, indicating that these amino acids may be substrates of YejA (69). Together, these data suggest that the YejABEF transporter of S. meliloti has a transport role that is important for growth in free-living bacteria. However, this ABC transporter has never been analyzed before in detail in the context of the rhizobium-legume symbiosis. Furthermore, even though this transporter is highly conserved among proteobacteria, its physiological role in whichever bacterium has been characterized in only a few instances. One of them is the uptake of microcin C in E. coli. This translation-inhibiting peptide-nucleotide of bacterial origin has no action on the bacterial membrane but has an intracellular target, the Asp aminoacyl-tRNA synthase (70). A strain with a mutation in the YejABEF transporter cannot take up microcin C and is resistant to it (71, 72). Conversely, the Salmonella and Brucella *yejABEF* mutants are more sensitive to peptides with membrane-damaging activities, such as defensins, polymyxin B, protamine, and melittin. Consequently, these mutants have reduced pathogenicity because their capacity to survive in macrophage cell lines or in mice is diminished (73, 74). Thus, the increased sensitivity of the *yejA*, *yejE*, and *yejF* mutants of S. meliloti toward NCRs is consistent with these previous findings.

The characteristics of the YejABEF peptide transporter toward membrane-damaging peptides versus peptides with intracellular actions are intriguingly parallel to the features of the BacA peptide transporter (named SbmA in E. coli and Salmonella). These transporters in E. coli, Salmonella, or S. meliloti are required for the import of diverse peptides with intracellular targets (microcins B17 and J25, Bac7, Bac5, and bleomycin), and mutants are therefore resistant to them, while the same mutants are hypersensitive to membrane-active peptides (defensins, NCRs) (19, 75–78). Moreover, the ranges of peptides that can be imported overlap between these two transporters. Both, YejABEF and SbmA in E. coli can take up microcin C derivatives (71). Furthermore, we show that in S. meliloti, both BacA and YejABEF contribute to NCR247 uptake. Intriguingly, our genetic analysis suggests that both transporters cooperate to import this peptide, since inactivation of one or the other abolishes or strongly reduces uptake. How these two transporters can physically interact and cooperate is an issue of interest for future research. Nevertheless, BacA can function (partially) in the absence of YejABEF, while the inverse is not the case. This may be the basis for the markedly different symbiotic phenotype of the *bacA* and *yej* mutants. While the *bacA* mutation is detrimental from the earliest contact with NCRs, and the mutant bacteria die as soon as they are released from infection threads in the symbiotic cells, the *yej* mutants cope with the NCR peptides much longer and show abnormalities only at the end of the bacteroid differentiation process and symbiont life span. The *yejA* mutant had a different NCR sensitivity profile than the *yejE* and *yejF* mutants, and this was correlated with a different symbiotic phenotype in the *M. sativa* host. This suggests that the periplasmic binding protein YejA contributes to the interaction with only a subset of the NCR peptides. Nevertheless, YejA seems to contribute to NCR247 uptake to an extent similar to that of YejE and YejF, despite the fact that, unlike with the *yejE* and *yejF* mutants, the sensitivity of the *yejA* mutant to the NCR247 peptide is not affected compared to that of the wild type.

How do the YejABEF and BacA transporters contribute to resistance to NCRs and other membrane-damaging peptides? The most straightforward model that has been proposed before for BacA (19–21), as well as for the unrelated SapABCDF peptide uptake transporter in Salmonella (79), is the reduction of the AMP concentration in the vicinity of the inner membrane below a critical threshold. Alternatively, the presence or activity of the transporters might indirectly affect the bacterial envelope structure, rendering it more robust against AMPs. The higher sensitivity of the *yej* mutants toward SDS is in agreement with this possibility. Similarly, membrane alterations were reported in the *bacA* mutant of S. meliloti (80).

### Conclusions.

The multifaceted NCR resistance required for symbiosis and chronic infection of the nodule cells mirrors the multitude of AMP resistance mechanisms in animal pathogens, which collectively contribute to the pathogenicity of these bacteria (2, 3, 81). However, one dimension of this strategy in pathogens is not known in S. meliloti and consists of the direct recognition of host AMPs by receptors triggering an adaptive response. Probably the best-studied AMP receptor is the two-component regulator PhoPQ in Salmonella, which adjusts the LPS composition in response to the presence of host peptides (64). In this respect, it is of interest to note that the rhizobial LPS structure changes strongly in bacteroids of NCR-producing nodules (82, 83). Perhaps the S. meliloti ExoS-ChvI or FeuP-FeuQ two-component regulators, which are upregulated by NCR treatment and essential for symbiosis (26, 46, 47), form such a regulatory module, recognizing NCRs and controlling an appropriate response in the bacteroids. Strikingly, the *yejA* promoter is a proposed direct target of ChvI (84).

As shown here, rhizobia have to defend themselves to be able to establish a chronic infection in the NCR-producing symbiotic cells of the nodules. On the other hand, the profile of NCR peptides produced in the nodule cells is also determinant for the outcome of the symbiosis, and some *M. truncatula* strains with mutations in individual *NCR* genes or *M. truncatula* strains expressing specific *NCR* alleles display incompatibility with S. meliloti strains (85–88). Thus, a fine balance must be established in the symbiotic nodule cells between the NCR landscapes and matching multifactorial bacterial countermeasures. Perturbations in the host or in the endosymbiont, like the ones described here, affecting this equilibrium lead to a breakdown of the symbiosis.

## MATERIALS AND METHODS

### Bacterial strains, plant growth and nodulation assays, and analysis.

The procedures for the growth of the S. meliloti Sm1021 strain and its derivatives (see Table S1 in the supplemental material) and plant culture of the *M. sativa* cultivar Gabès, the *M. truncatula* accession strain Jemalong A17, and the A17 *dnf1* mutant, as well as nodulation assays, acetylene reduction assays, bacteroid isolation, flow cytometry measurements, and confocal microscopy, were performed as described before (89).

Bacterial mutants with mutations in *rpoH1*, *yejA*, *yejE*, and *yejF* were obtained by plasmid insertion or by gene deletions as described before (90). For plasmid insertion mutagenesis, PCR-amplified internal gene fragments were cloned into the pVO155-p*npt2*::*GFP* plasmid (Table S1), followed by cointegration of the resulting construct in the S. meliloti genome. Deletion mutants of complete open reading frames were obtained by double crossover of the plasmid pNPTS129, carrying the PCR-amplified merged upstream and downstream regions of the target gene (Table S1). Constructs in the pVO155-p*npt2*::*GFP* and pNPTS129 plasmids were transferred from E. coli into S. meliloti by triparental conjugation using the helper plasmid pRK600 (Table S1).

For complementation studies via merodiploid strains, a DNA fragment containing the *yejABEF* genes was PCR amplified from S. meliloti Sm1021 genomic DNA using primers yejA_NdeI_F and yejF_XbaI_R (Table S1) and cloned between the NdeI and XbaI restriction sites of the pSRKGm vector (91). The resulting construct, pSRK-*yejABEF* (Table S1), was confirmed by restriction analysis and sequencing. The plasmid and the empty pSRKGm vector were conjugated to wild-type Sm1021 or the *yejE* and *yejF* deletion mutants by triparental mating. In the pSRK-*yejABEF* plasmid, the *yejABEF* genes are downstream of the *lac* promoter, providing a low basal expression and an isopropyl-β-d-thiogalactopyranoside (IPTG)-inducible expression. To ensure sufficient expression of the plasmid-derived *yejABEF* genes in the nodules infected with strains carrying the pSRK-*yejABEF* plasmid, plants were watered twice per week with 50 ml 1 mM IPTG-containing nutrient solution.

Derivatives of the S. meliloti wild-type strain and the *bacA*, *yejE*, and *yejF* mutants, carrying a *GFP* gene under the control of the *nifH* promoter on the broad-host-range, low-copy-number IncP plasmid pHC60-p*nifH*::*GFP*, were obtained by triparental mating (Table S1).

The *in vitro* sensitivity assays (Fig. 1 and 6; Fig. S2) were performed with the *yejA*, *yejE*, and *yejF* plasmid insertion mutants. Initial nodulation experiments were performed with both the *yejA*, *yejE*, and *yejF* plasmid insertion mutants and the corresponding deletion mutants. Since both types of mutants displayed identical phenotypes in symbiosis, all subsequent nodulation experiments, including all experiments shown here, were performed with the deletion mutants.

Quantitative analysis of the shape of free-living bacteria and bacteroids was performed with the MicrobeJ plugin of ImageJ (92). Bacteroid extracts and exponential-phase cultures were stained with 2.5 nM SYTO 9 for 10 min at 37°C and mounted between slides and coverslips. Bacterial imaging was performed on an SP8 laser-scanning confocal microscope (Leica Microsystems) equipped with hybrid detectors and a 63× oil immersion objective (Plan Apo, numerical aperture [NA], 1.4; Leica). For each condition, multiple z-stacks (2.7-μm width, 0.7-μm step) were automatically acquired (excitation, 488 nm; collection of fluorescence, 520 to 580 nm). Stacks were transformed as maximum intensity projections using ImageJ software (93). Bacteria in the image stacks were automatically detected with MicrobeJ using an intensity-based threshold method with a combination of morphological filters. To ensure high data quality, every image was manually checked to remove false-positive objects (mainly plant cell debris). Morphological parameters were directly extracted from MicrobeJ, and figures were created with Excel or ggplot2 in R.

### *In vitro* sensitivity and peptide uptake assays.

NCR sensitivity assays were carried out essentially as described previously (20). Triplicate measurements were performed in at least two independent experiments per treatment. Nonparametric Kruskal-Wallis and *post hoc* Dunn tests were performed to assess the significance of differences between the sensitivities of the wild type and the mutant strains. To measure the resistance of strains to SDS, H_2_O_2_, and HCl stress, overnight cultures of the wild type and mutants were diluted to an optical density at 600 nm (OD_600_) of 0.2. A total of 100 μl of these suspensions was added to 3 ml soft agar (0.7% agar) and poured onto 1.5% agar plates. After solidification of the soft agar, filter paper disks (5-mm diameter) were placed on the center of the plate, and 5 μl of 10% (wt/vol) SDS, 2 M HCl, or 1% H_2_O_2_ was added to the disks. Plates were incubated at 28°C for 3 days, and the diameter of the clearing zone was measured. For microaerobic and anaerobic treatments, cultures of the wild type and mutants were diluted to an OD_600_ of 0.1, and 5-fold dilution series of each were prepared. The dilution series were subsequently spotted (5 μl per spot) on agar plates. One series of plates was grown under aerobic conditions. A second series of plates was placed in a 2.5-liter airtight jar containing an AnaeroPack-MicroAero (Mitsubishi Gas Chemical) bag for microaerobic growth (6 to 12% O_2_, according to the product specifications). The third series of plates was placed in a 2.5-liter jar containing an AnaeroGen 2.5-liter (Thermo Scientific) bag for anaerobic growth (<0.1% O_2_, according to the product specifications). All plates were grown at 28°C, and after 3 days, the plates were removed from the jars for the microaerobic and anaerobic conditions. After 3 days, colonies were sufficiently grown under the aerobic condition for counting. Growth was observed under the microaerobic conditions but was less strong than under the aerobic condition, and these plates were further incubated for 1 day before colony counting. No growth was observed after 3 days under the anaerobic conditions, and plates were incubated for 4 days under aerobic conditions before colony counting. Colony counting was done using a binocular. Quadruplicate measurements and two independent experiments per treatment were performed. Nonparametric Kruskal-Wallis and *post hoc* Dunn tests were performed to assess significance of differences in all the assays. For growth curves, precultures were diluted to an OD_600_ of 0.04 in YEB medium (89). Cultures were dispatched in microtiter plates with 200 μl of culture per well. The plates were incubated in a SPECTROstar Nano (BMG Labtech) plate reader for 100 h at 28°C with 100-rpm shaking, and OD_600_ measurements were taken every 30 min. Doubling times were calculated from the obtained growth curves (*n* = 10), and nonparametric Kruskal-Wallis and *post hoc* Dunn tests were performed to assess the significance of differences between growth rates. Peptide uptake assays were performed in quadruplicate as described previously (20, 94).

### TEM.

OD_600_ bacterial suspensions of 6- or 21-day-old nodule samples were incubated in fixative (3% glutaraldehyde, 1% paraformaldehyde in 0.1 M cacodylate, pH 6.8) for 1 to 3 h and washed with 0.1 M cacodylate buffer, pH 6.8. Samples were then incubated for 1 h in 1% osmium tetroxide, 1.5% potassium ferrocyanide in water. After being washed, bacterial suspensions were pelleted in 2% low-melting-point agarose to facilitate their manipulation. Samples were dehydrated by incubation in increasing concentrations of ethanol (nodule samples for a total of 4 h in 10, 20, 30, 50, 70, 90, and 100% absolute ethanol-propylene oxide; bacterial pellets for a total of 2 h in 10, 30, 50, 70, 90, and 100% absolute ethanol), followed by infiltration with epoxy resin (low-viscosity Premix kit medium; Agar Scientific) (for nodule samples, 3 days of infiltration; for bacterial pellets, 24 h of infiltration) and polymerization for 24 h at 60°C. Ultrathin sections (80 to 70 nm) were obtained with an ultramicrotome EM UC6 (Leica Microsystems) and collected on Formvar carbon-coated copper grids (Agar Scientific). Sections were stained with 2% uranyl acetate (Merck) and lead citrate (Agar Scientific) before being observed with a JEOL JEM-1400 transmission electron microscope operating at 120 kV. Images were acquired using a post-column chromatography high-resolution (11-megapixel) high-speed camera (SC1000 Orius; Gatan) and processed with Digital Micrograph (Gatan).

## References

[1] Hanson MA, Lemaitre B. 2020. New insights on *Drosophila* antimicrobial peptide function in host defense, and beyond. Curr Opin Immunol 62:22–30. doi:10.1016/j.coi.2019.11.008. 31835066

[2] Mergaert P. 2018. Role of antimicrobial peptides in controlling symbiotic bacterial populations. Nat Prod Rep 35:336–356. doi:10.1039/c7np00056a. 29393944

[3] Maróti G, Kereszt A, Kondorosi E, Mergaert P. 2011. Natural roles of antimicrobial peptides in microbes, plants and animals. Res Microbiol 162:363–374. doi:10.1016/j.resmic.2011.02.005. 21320593

[4] Mergaert P, Kikuchi Y, Shigenobu S, Nowack ECM. 2017. Metabolic integration of bacterial endosymbionts through antimicrobial peptides. Trends Microbiol 25:703–712. doi:10.1016/j.tim.2017.04.007. 28549825

[5] Kondorosi E, Mergaert P, Kereszt A. 2013. A paradigm for endosymbiotic life: cell differentiation of *Rhizobium* bacteria provoked by host plant factors. Annu Rev Microbiol 67:611–628. doi:10.1146/annurev-micro-092412-155630. 24024639

[6] Alunni B, Gourion B. 2016. Terminal bacteroid differentiation in the legume-rhizobium symbiosis: nodule-specific cysteine-rich peptides and beyond. New Phytol 211:411–417. doi:10.1111/nph.14025. 27241115

[7] Pan H, Wang D. 2017. Nodule cysteine-rich peptides maintain a working balance during nitrogen-fixing symbiosis. Nat Plants 3:17048. doi:10.1038/nplants.2017.48. 28470183

[8] Stonoha-Arther C, Wang D. 2018. Tough love: accommodating intracellular bacteria through directed secretion of antimicrobial peptides during the nitrogen-fixing symbiosis. Curr Opin Plant Biol 44:155–163. doi:10.1016/j.pbi.2018.04.017. 29778978

[9] Roy P, Achom M, Wilkinson H, Lagunas B, Gifford ML. 2020. Symbiotic outcome modified by the diversification from 7 to over 700 nodule-specific cysteine-rich peptides. Genes 11:348. doi:10.3390/genes11040348. PMC7230169 32218172

[10] Mergaert P, Uchiumi T, Alunni B, Evanno G, Cheron A, Catrice O, Mausset A-E, Barloy-Hubler F, Galibert F, Kondorosi A, Kondorosi E. 2006. Eukaryotic control on bacterial cell cycle and differentiation in the *Rhizobium*-legume symbiosis. Proc Natl Acad Sci U S A 103:5230–5235. doi:10.1073/pnas.0600912103. 16547129 PMC1458823

[11] Czernic P, Gully D, Cartieaux F, Moulin L, Guefrachi I, Patrel D, Pierre O, Fardoux J, Chaintreuil C, Nguyen P, Gressent F, Da Silva C, Poulain J, Wincker P, Rofidal V, Hem S, Barrière Q, Arrighi J-F, Mergaert P, Giraud E. 2015. Convergent evolution of endosymbiont differentiation in Dalbergioid and Inverted Repeat-Lacking Clade legumes mediated by nodule-specific cysteine-rich peptides. Plant Physiol 169:1254–1265. doi:10.1104/pp.15.00584. 26286718 PMC4587450

[12] Mergaert P, Nikovics K, Kelemen Z, Maunoury N, Vaubert D, Kondorosi A, Kondorosi E. 2003. A novel family in *Medicago truncatula* consisting of more than 300 nodule-specific genes coding for small, secreted polypeptides with conserved cysteine motifs. Plant Physiol 132:161–173. doi:10.1104/pp.102.018192. 12746522 PMC166962

[13] Van de Velde W, Zehirov G, Szatmari A, Debreczeny M, Ishihara H, Kevei Z, Farkas A, Mikulass K, Nagy A, Tiricz H, Satiat-Jeunemaître B, Alunni B, Bourge M, Kucho K, Abe M, Kereszt A, Maroti G, Uchiumi T, Kondorosi E, Mergaert P. 2010. Plant peptides govern terminal differentiation of bacteria in symbiosis. Science 327:1122–1126. doi:10.1126/science.1184057. 20185722

[14] Montiel J, Downie JA, Farkas A, Bihari P, Herczeg R, Bálint B, Mergaert P, Kereszt A, Kondorosi É. 2017. Morphotype of bacteroids in different legumes correlates with the number and type of symbiotic NCR peptides. Proc Natl Acad Sci U S A 114:5041–5046. doi:10.1073/pnas.1704217114. 28438996 PMC5441718

[15] Guefrachi I, Nagymihaly M, Pislariu CI, Van de Velde W, Ratet P, Mars M, Udvardi MK, Kondorosi E, Mergaert P, Alunni B. 2014. Extreme specificity of *NCR* gene expression in *Medicago truncatula*. BMC Genomics 15:712. doi:10.1186/1471-2164-15-712. 25156206 PMC4168050

[16] Lima RM, Kylarová S, Mergaert P, Kondorosi É. 2020. Unexplored arsenals of legume peptides with potential for their applications in medicine and agriculture. Front Microbiol 11:1307. doi:10.3389/fmicb.2020.01307. 32625188 PMC7314904

[17] Mikuláss KR, Nagy K, Bogos B, Szegletes Z, Kovács E, Farkas A, Váró G, Kondorosi É, Kereszt A. 2016. Antimicrobial nodule-specific cysteine-rich peptides disturb the integrity of bacterial outer and inner membranes and cause loss of membrane potential. Ann Clin Microbiol Antimicrob 15:43. doi:10.1186/s12941-016-0159-8. 27465344 PMC4964015

[18] Farkas A, Maróti G, Durgő H, Györgypál Z, Lima RM, Medzihradszky KF, Kereszt A, Mergaert P, Kondorosi É. 2014. *Medicago truncatula* symbiotic peptide NCR247 contributes to bacteroid differentiation through multiple mechanisms. Proc Natl Acad Sci U S A 111:5183–5188. doi:10.1073/pnas.1404169111. 24706863 PMC3986156

[19] Haag AF, Baloban M, Sani M, Kerscher B, Pierre O, Farkas A, Longhi R, Boncompagni E, Hérouart D, Dall'angelo S, Kondorosi E, Zanda M, Mergaert P, Ferguson GP. 2011. Protection of *Sinorhizobium* against host cysteine-rich antimicrobial peptides is critical for symbiosis. PLoS Biol 9:e1001169. doi:10.1371/journal.pbio.1001169. 21990963 PMC3186793

[20] Barrière Q, Guefrachi I, Gully D, Lamouche F, Pierre O, Fardoux J, Chaintreuil C, Alunni B, Timchenko T, Giraud E, Mergaert P. 2017. Integrated roles of BclA and dd-carboxypeptidase 1 in Bradyrhizobium differentiation within NCR-producing and NCR-lacking root nodules. Sci Rep 7:9063. doi:10.1038/s41598-017-08830-0. 28831061 PMC5567381

[21] Guefrachi I, Pierre O, Timchenko T, Alunni B, Barrière Q, Czernic P, Villaécija-Aguilar J-A, Verly C, Bourge M, Fardoux J, Mars M, Kondorosi E, Giraud E, Mergaert P. 2015. *Bradyrhizobium* BclA is a peptide transporter required for bacterial differentiation in symbiosis with *Aeschynomene* legumes. Mol Plant Microbe Interact 28:1155–1166. doi:10.1094/MPMI-04-15-0094-R. 26106901

[22] Rempel S, Gati C, Nijland M, Thangaratnarajah C, Karyolaimos A, de Gier JW, Guskov A, Slotboom DJ. 2020. A mycobacterial ABC transporter mediates the uptake of hydrophilic compounds. Nature 580:409–412. doi:10.1038/s41586-020-2072-8. 32296172

[23] Arnold MFF, Penterman J, Shabab M, Chen EJ, Walker GC. 2018. Important late-stage symbiotic role of the *Sinorhizobium meliloti* exopolysaccharide succinoglycan. J Bacteriol 200:e00665-17. doi:10.1128/JB.00665-17. 29632097 PMC5996692

[24] Cole JN, Nizet V. 2016. Bacterial evasion of host antimicrobial peptide defenses. Microbiol Spectr 4:VMBF-0006-2015. doi:10.1128/microbiolspec.VMBF-0006-2015. PMC4804471 26999396

[25] Arnold MFF, Shabab M, Penterman J, Boehme KL, Griffitts JS, Walker GC. 2017. Genome-wide sensitivity analysis of the microsymbiont *Sinorhizobium meliloti* to symbiotically important, defensin-like host peptides. mBio 8:e01060-17. doi:10.1128/mBio.01060-17. 28765224 PMC5539429

[26] Penterman J, Abo RP, De Nisco NJ, Arnold MFF, Longhi R, Zanda M, Walker GC. 2014. Host plant peptides elicit a transcriptional response to control the *Sinorhizobium meliloti* cell cycle during symbiosis. Proc Natl Acad Sci U S A 111:3561–3566. doi:10.1073/pnas.1400450111. 24501120 PMC3948309

[27] Tiricz H, Szucs A, Farkas A, Pap B, Lima RM, Maróti G, Kondorosi É, Kereszt A. 2013. Antimicrobial nodule-specific cysteine-rich peptides induce membrane depolarization-associated changes in the transcriptome of *Sinorhizobium meliloti*. Appl Environ Microbiol 79:6737–6746. doi:10.1128/AEM.01791-13. 23995935 PMC3811505

[28] Flores-Tinoco CE, Tschan F, Fuhrer T, Margot C, Sauer U, Christen M, Christen B. 2020. Co-catabolism of arginine and succinate drives symbiotic nitrogen fixation. Mol Syst Biol 16:e9419. doi:10.15252/msb.20199419. 32490601 PMC7268258

[29] Simpson BW, Trent MS. 2019. Pushing the envelope: LPS modifications and their consequences. Nat Rev Microbiol 17:403–416. doi:10.1038/s41579-019-0201-x. 31142822 PMC6913091

[30] Campbell GRO, Sharypova LA, Scheidle H, Jones KM, Niehaus K, Becker A, Walker GC. 2003. Striking complexity of lipopolysaccharide defects in a collection of *Sinorhizobium meliloti* mutants. J Bacteriol 185:3853–3862. doi:10.1128/JB.185.13.3853-3862.2003. 12813079 PMC161594

[31] Haag AF, Wehmeier S, Beck S, Marlow VL, Fletcher V, James EK, Ferguson GP. 2009. The *Sinorhizobium meliloti* LpxXL and AcpXL proteins play important roles in bacteroid development within alfalfa. J Bacteriol 191:4681–4686. doi:10.1128/JB.00318-09. 19429615 PMC2704725

[32] Barnett MJ, Bittner AN, Toman CJ, Oke V, Long SR. 2012. Dual RpoH sigma factors and transcriptional plasticity in a symbiotic bacterium. J Bacteriol 194:4983–4994. doi:10.1128/JB.00449-12. 22773790 PMC3430346

[33] Mitsui H, Sato T, Sato Y, Ito N, Minamisawa K. 2004. *Sinorhizobium meliloti* RpoH1 is required for effective nitrogen-fixing symbiosis with alfalfa. Mol Genet Genomics 271:416–425. doi:10.1007/s00438-004-0992-x. 15007732

[34] Oke V, Rushing BG, Fisher EJ, Moghadam-Tabrizi M, Long SR. 2001. Identification of the heat-shock sigma factor RopH and a second RpoH-like protein in *Sinorhizobium meliloti*. Microbiology (Reading) 147:2399–2408. doi:10.1099/00221287-147-9-2399. 11535780

[35] Ono Y, Mitsui H, Sato T, Minamisawa K. 2001. Two RpoH homologs responsible for the expression of heat shock protein genes in Sinorhizobium meliloti. Mol Gen Genet 264:902–912. doi:10.1007/s004380000380. 11254138

[36] Roux B, Rodde N, Jardinaud M-F, Timmers T, Sauviac L, Cottret L, Carrère S, Sallet E, Courcelle E, Moreau S, Debellé F, Capela D, de Carvalho-Niebel F, Gouzy J, Bruand C, Gamas P. 2014. An integrated analysis of plant and bacterial gene expression in symbiotic root nodules using laser-capture microdissection coupled to RNA sequencing. Plant J 77:817–837. doi:10.1111/tpj.12442. 24483147

[37] Santos R, Hérouart D, Sigaud S, Touati D, Puppo A. 2001. Oxidative burst in alfalfa-*Sinorhizobium meliloti* symbiotic interaction. Mol Plant Microbe Interact 14:86–89. doi:10.1094/MPMI.2001.14.1.86. 11194876

[38] Pierre O, Engler G, Hopkins J, Brau F, Boncompagni E, Hérouart D. 2013. Peribacteroid space acidification: a marker of mature bacteroid functioning in *Medicago truncatula* nodules. Plant Cell Environ 36:2059–2070. doi:10.1111/pce.12116. 23586685

[39] Soupène E, Foussard M, Boistard P, Truchet G, Batut J. 1995. Oxygen as a key developmental regulator of *Rhizobium meliloti* N_2_-fixation gene expression within the alfalfa root nodule. Proc Natl Acad Sci U S A 92:3759–3763. doi:10.1073/pnas.92.9.3759. 7731979 PMC42041

[40] de Lucena DK, Pühler A, Weidner S. 2010. The role of sigma factor RpoH1 in the pH stress response of *Sinorhizobium meliloti*. BMC Microbiol 10:265. doi:10.1186/1471-2180-10-265. 20955556 PMC2976971

[41] Ferguson GP, Datta A, Carlson RW, Walker GC. 2005. Importance of unusually modified lipid A in *Sinorhizobium* stress resistance and legume symbiosis. Mol Microbiol 56:68–80. doi:10.1111/j.1365-2958.2005.04536.x. 15773979

[42] Niehaus K, Lagares A, Pühler A. 1998. A *Sinorhizobium meliloti* lipopolysaccharide mutant induces effective nodules on the host plant *Medicago sativa* (alfalfa) but fails to establish a symbiosis with *Medicago truncatula*. MPMI 11:906–914. doi:10.1094/MPMI.1998.11.9.906.

[43] Campbell GRO, Reuhs BL, Walker GC. 2002. Chronic intracellular infection of alfalfa nodules by *Sinorhizobium meliloti* requires correct lipopolysaccharide core. Proc Natl Acad Sci U S A 99:3938–3943. doi:10.1073/pnas.062425699. 11904442 PMC122627

[44] Wang D, Griffitts J, Starker C, Fedorova E, Limpens E, Ivanov S, Bisseling T, Long S. 2010. A nodule-specific protein secretory pathway required for nitrogen-fixing symbiosis. Science 327:1126–1129. doi:10.1126/science.1184096. 20185723 PMC4824053

[45] Mendoza-Suárez MA, Geddes BA, Sánchez-Cañizares C, Ramírez-González RH, Kirchhelle C, Jorrin B, Poole PS. 2020. Optimizing *Rhizobium*-legume symbioses by simultaneous measurement of rhizobial competitiveness and N_2_ fixation in nodules. Proc Natl Acad Sci U S A 117:9822–9831. doi:10.1073/pnas.1921225117. 32317381 PMC7211974

[46] Bélanger L, Dimmick KA, Fleming JS, Charles TC. 2009. Null mutations in *Sinorhizobium meliloti exoS* and *chvI* demonstrate the importance of this two-component regulatory system for symbiosis. Mol Microbiol 74:1223–1237. doi:10.1111/j.1365-2958.2009.06931.x. 19843226

[47] Griffitts JS, Carlyon RE, Erickson JH, Moulton JL, Barnett MJ, Toman CJ, Long SR. 2008. A *Sinorhizobium meliloti* osmosensory two-component system required for cyclic glucan export and symbiosis. Mol Microbiol 69:479–490. doi:10.1111/j.1365-2958.2008.06304.x. 18630344

[48] Santos R, Hérouart D, Puppo A, Touati D. 2000. Critical protective role of bacterial superoxide dismutase in rhizobium-legume symbiosis. Mol Microbiol 38:750–759. doi:10.1046/j.1365-2958.2000.02178.x. 11115110

[49] Jamet A, Sigaud S, Van de Sype G, Puppo A, Hérouart D. 2003. Expression of the bacterial catalase genes during *Sinorhizobium meliloti*-*Medicago sativa* symbiosis and their crucial role during the infection process. Mol Plant Microbe Interact 16:217–225. doi:10.1094/MPMI.2003.16.3.217. 12650453

[50] Castro-Sowinski S, Matan O, Bonafede P, Okon Y. 2007. A thioredoxin of *Sinorhizobium meliloti* CE52G is required for melanin production and symbiotic nitrogen fixation. Mol Plant Microbe Interact 20:986–993. doi:10.1094/MPMI-20-8-0986. 17724847

[51] Benyamina SM, Baldacci-Cresp F, Couturier J, Chibani K, Hopkins J, Bekki A, de Lajudie P, Rouhier N, Jacquot J-P, Alloing G, Puppo A, Frendo P. 2013. Two Sinorhizobium meliloti glutaredoxins regulate iron metabolism and symbiotic bacteroid differentiation. Environ Microbiol 15:795–810. doi:10.1111/j.1462-2920.2012.02835.x. 22891731

[52] Tang G, Li N, Liu Y, Yu L, Yan J, Luo L. 2018. *Sinorhizobium meliloti* glutathione reductase is required for both redox homeostasis and symbiosis. Appl Environ Microbiol 84:e01937-17. doi:10.1128/AEM.01937-17. PMC5772252 29150514

[53] Yang L, El Msehli S, Benyamina S, Lambert A, Hopkins J, Cazareth J, Pierre O, Hérouart D, Achi-Smiti S, Boncompagni E, Frendo P. 2020. Glutathione deficiency in *Sinorhizobium meliloti* does not impair bacteroid differentiation but induces early senescence in the interaction with *Medicago truncatula*. Front Plant Sci 11:137. doi:10.3389/fpls.2020.00137. 32194584 PMC7063052

[54] Tang G, Xing S, Wang S, Yu L, Li X, Staehelin C, Yang M, Luo L. 2017. Regulation of cysteine residues in LsrB proteins from *Sinorhizobium meliloti* under free-living and symbiotic oxidative stress. Environ Microbiol 19:5130–5145. doi:10.1111/1462-2920.13992. 29124841

[55] Bittner AN, Foltz A, Oke V. 2007. Only one of five *groEL* genes is required for viability and successful symbiosis in *Sinorhizobium meliloti*. J Bacteriol 189:1884–1889. doi:10.1128/JB.01542-06. 17158666 PMC1855696

[56] Ogden AJ, McAleer JM, Kahn ML. 2019. Characterization of the *Sinorhizobium meliloti* HslUV and ClpXP protease systems in free-living and symbiotic states. J Bacteriol 201:e00498-18. doi:10.1128/JB.00498-18. 30670545 PMC6416911

[57] Cosme AM, Becker A, Santos MR, Sharypova LA, Santos PM, Moreira LM. 2008. The outer membrane protein TolC from *Sinorhizobium meliloti* affects protein secretion, polysaccharide biosynthesis, antimicrobial resistance, and symbiosis. Mol Plant Microbe Interact 21:947–957. doi:10.1094/MPMI-21-7-0947. 18533835

[58] Gruber TM, Gross CA. 2003. Multiple sigma subunits and the partitioning of bacterial transcription space. Annu Rev Microbiol 57:441–466. doi:10.1146/annurev.micro.57.030502.090913. 14527287

[59] Nonaka G, Blankschien M, Herman C, Gross CA, Rhodius VA. 2006. Regulon and promoter analysis of the *E. coli* heat-shock factor, sigma32, reveals a multifaceted cellular response to heat stress. Genes Dev 20:1776–1789. doi:10.1101/gad.1428206. 16818608 PMC1522074

[60] Guest RL, Raivio TL. 2016. Role of the gram-negative envelope stress response in the presence of antimicrobial agents. Trends Microbiol 24:377–390. doi:10.1016/j.tim.2016.03.001. 27068053

[61] Sauviac L, Philippe H, Phok K, Bruand C. 2007. An extracytoplasmic function sigma factor acts as a general stress response regulator in *Sinorhizobium meliloti*. J Bacteriol 189:4204–4216. doi:10.1128/JB.00175-07. 17400745 PMC1913381

[62] Lang C, Barnett MJ, Fisher RF, Smith LS, Diodati ME, Long SR. 2018. Most S*inorhizobium meliloti* extracytoplasmic function sigma factors control accessory functions. mSphere 3:e00454-18. doi:10.1128/mSphereDirect.00454-18. 30305320 PMC6180224

[63] Zasloff M. 2002. Antimicrobial peptides of multicellular organisms. Nature 415:389–395. doi:10.1038/415389a. 11807545

[64] Dalebroux ZD, Miller SI. 2014. *Salmonellae* PhoPQ regulation of the outer membrane to resist innate immunity. Curr Opin Microbiol 17:106–113. doi:10.1016/j.mib.2013.12.005. 24531506 PMC4043142

[65] Lagares A, Hozbor DF, Niehaus K, Otero AJ, Lorenzen J, Arnold W, Pühler A. 2001. Genetic characterization of a *Sinorhizobium meliloti* chromosomal region in lipopolysaccharide biosynthesis. J Bacteriol 183:1248–1258. doi:10.1128/JB.183.4.1248-1258.2001. 11157937 PMC94998

[66] Jardinaud M-F, Boivin S, Rodde N, Catrice O, Kisiala A, Lepage A, Moreau S, Roux B, Cottret L, Sallet E, Brault M, Emery RJN, Gouzy J, Frugier F, Gamas P. 2016. A laser dissection-RNAseq analysis highlights the activation of cytokinin pathways by Nod factors in the *Medicago truncatula* root epidermis. Plant Physiol 171:2256–2276. doi:10.1104/pp.16.00711. 27217496 PMC4936592

[67] Liu C-W, Breakspear A, Guan D, Cerri MR, Jackson K, Jiang S, Robson F, Radhakrishnan GV, Roy S, Bone C, Stacey N, Rogers C, Trick M, Niebel A, Oldroyd GED, de Carvalho-Niebel F, Murray JD. 2019. NIN acts as a network hub controlling a growth module required for rhizobial infection. Plant Physiol 179:1704–1722. doi:10.1104/pp.18.01572. 30710053 PMC6446755

[68] diCenzo GC, Benedict AB, Fondi M, Walker GC, Finan TM, Mengoni A, Griffitts JS. 2018. Robustness encoded across essential and accessory replicons of the ecologically versatile bacterium *Sinorhizobium meliloti*. PLoS Genet 14:e1007357. doi:10.1371/journal.pgen.1007357. 29672509 PMC5929573

[69] Mauchline TH, Fowler JE, East AK, Sartor AL, Zaheer R, Hosie AH, Poole PS, Finan TM. 2006. Mapping the *Sinorhizobium meliloti* 1021 solute-binding protein-dependent transportome. Proc Natl Acad Sci U S A 103:17933–17938. doi:10.1073/pnas.0606673103. 17101990 PMC1635973

[70] Travin DY, Severinov K, Dubiley S. 2021. Natural Trojan horse inhibitors of aminoacyl-tRNA sythetases. RSC Chem Biol 2021:468–485. doi:10.1039/D0CB00208A. PMC8323819 34382000

[71] Bantysh O, Serebryakova M, Zukher I, Kulikovsky A, Tsibulskaya D, Dubiley S, Severinov K. 2015. Enzymatic synthesis and functional characterization of bioactive microcin C-like compounds with altered peptide sequence and length. J Bacteriol 197:3133–3141. doi:10.1128/JB.00271-15. 26195597 PMC4560285

[72] Novikova M, Metlitskaya A, Datsenko K, Kazakov T, Kazakov A, Wanner B, Severinov K. 2007. The *Escherichia coli* Yej transporter is required for the uptake of translation inhibitor microcin C. J Bacteriol 189:8361–8365. doi:10.1128/JB.01028-07. 17873039 PMC2168686

[73] Eswarappa SM, Panguluri KK, Hensel M, Chakravortty D. 2008. The yejABEF operon of Salmonella confers resistance to antimicrobial peptides and contributes to its virulence. Microbiology (Reading) 154:666–678. doi:10.1099/mic.0.2007/011114-0. 18227269

[74] Wang Z, Bie P, Cheng J, Lu L, Cui B, Wu Q. 2016. The ABC transporter YejABEF is required for resistance to antimicrobial peptides and the virulence of *Brucella melitensis*. Sci Rep 6:31876. doi:10.1038/srep31876. 27550726 PMC4994006

[75] Salomón RA, Farías RN. 1995. The peptide antibiotic microcin 25 is imported through the TonB pathway and the SbmA protein. J Bacteriol 177:3323–3325. doi:10.1128/jb.177.11.3323-3325.1995. 7768835 PMC177028

[76] Laviña M, Pugsley AP, Moreno F. 1986. Identification, mapping, cloning and characterization of a gene (sbmA) required for microcin B17 action on *Escherichia coli* K12. J Gen Microbiol 132:1685–1693. doi:10.1099/00221287-132-6-1685. 3543211

[77] Wehmeier S, Arnold MFF, Marlow VL, Aouida M, Myka KK, Fletcher V, Benincasa M, Scocchi M, Ramotar D, Ferguson GP. 2010. Internalization of a thiazole-modified peptide in *Sinorhizobium meliloti* occurs by BacA-dependent and -independent mechanisms. Microbiology (Reading) 156:2702–2713. doi:10.1099/mic.0.039909-0. 20507886

[78] Arnold MFF, Haag AF, Capewell S, Boshoff HI, James EK, McDonald R, Mair I, Mitchell AM, Kerscher B, Mitchell TJ, Mergaert P, Barry CE, Scocchi M, Zanda M, Campopiano DJ, Ferguson GP. 2013. Partial complementation of *Sinorhizobium meliloti bacA* mutant phenotypes by the *Mycobacterium tuberculosis* BacA protein. J Bacteriol 195:389–398. doi:10.1128/JB.01445-12. 23161027 PMC3553841

[79] Parra-Lopez C, Baer MT, Groisman EA. 1993. Molecular genetic analysis of a locus required for resistance to antimicrobial peptides in *Salmonella typhimurium*. EMBO J 12:4053–4062. doi:10.1002/j.1460-2075.1993.tb06089.x. 8223423 PMC413698

[80] Ferguson GP, Roop RM, Walker GC. 2002. Deficiency of a *Sinorhizobium meliloti* BacA mutant in alfalfa symbiosis correlates with alteration of the cell envelope. J Bacteriol 184:5625–5632. doi:10.1128/JB.184.20.5625-5632.2002. 12270820 PMC139620

[81] Bauer ME, Shafer WM. 2015. On the *in vivo* significance of bacterial resistance to antimicrobial peptides. Biochim Biophys Acta 1848:3101–3111. doi:10.1016/j.bbamem.2015.02.012. 25701234 PMC4540701

[82] Kannenberg EL, Carlson RW. 2001. Lipid A and O-chain modifications cause *Rhizobium* lipopolysaccharides to become hydrophobic during bacteroid development. Mol Microbiol 39:379–391. doi:10.1046/j.1365-2958.2001.02225.x. 11136459

[83] Crespo-Rivas JC, Guefrachi I, Mok KC, Villaécija-Aguilar JA, Acosta-Jurado S, Pierre O, Ruiz-Sainz JE, Taga ME, Mergaert P, Vinardell JM. 2016. *Sinorhizobium fredii* HH103 bacteroids are not terminally differentiated and show altered O-antigen in nodules of the inverted repeat-lacking clade legume *Glycyrrhiza uralensis*. Environ Microbiol 18:2392–2404. doi:10.1111/1462-2920.13101. 26521863

[84] Ratib NR, Sabio EY, Mendoza C, Barnett MJ, Clover SB, Ortega JA, Dela Cruz FM, Balderas D, White H, Long SR, Chen EJ. 2018. Genome-wide identification of genes directly regulated by ChvI and a consensus sequence for ChvI binding in Sinorhizobium meliloti. Mol Microbiol 110:596–615. doi:10.1111/mmi.14119. 30192418 PMC6343485

[85] Kim M, Chen Y, Xi J, Waters C, Chen R, Wang D. 2015. An antimicrobial peptide essential for bacterial survival in the nitrogen-fixing symbiosis. Proc Natl Acad Sci U S A 112:15238–15243. doi:10.1073/pnas.1500123112. 26598690 PMC4679048

[86] Horváth B, Domonkos Á, Kereszt A, Szűcs A, Ábrahám E, Ayaydin F, Bóka K, Chen Y, Chen R, Murray JD, Udvardi MK, Kondorosi É, Kaló P. 2015. Loss of the nodule-specific cysteine rich peptide, NCR169, abolishes symbiotic nitrogen fixation in the *Medicago truncatula dnf7* mutant. Proc Natl Acad Sci U S A 112:15232–15237. doi:10.1073/pnas.1500777112. 26401023 PMC4679056

[87] Wang Q, Yang S, Liu J, Terecskei K, Ábrahám E, Gombár A, Domonkos Á, Szűcs A, Körmöczi P, Wang T, Fodor L, Mao L, Fei Z, Kondorosi É, Kaló P, Kereszt A, Zhu H. 2017. Host-secreted antimicrobial peptide enforces symbiotic selectivity in *Medicago truncatula*. Proc Natl Acad Sci U S A 114:6854–6859. doi:10.1073/pnas.1700715114. 28607058 PMC5495241

[88] Yang S, Wang Q, Fedorova E, Liu J, Qin Q, Zheng Q, Price PA, Pan H, Wang D, Griffitts JS, Bisseling T, Zhu H. 2017. Microsymbiont discrimination mediated by a host-secreted peptide in *Medicago truncatula*. Proc Natl Acad Sci U S A 114:6848–6853. doi:10.1073/pnas.1700460114. 28607056 PMC5495240

[89] Kazmierczak T, Nagymihály M, Lamouche F, Barrière Q, Guefrachi I, Alunni B, Ouadghiri M, Ibijbijen J, Kondorosi É, Mergaert P, Gruber V. 2017. Specific host-responsive associations between *Medicago truncatula* accessions and *Sinorhizobium* strains. Mol Plant Microbe Interact 30:399–409. doi:10.1094/MPMI-01-17-0009-R. 28437159

[90] Gully D, Gargani D, Bonaldi K, Grangeteau C, Chaintreuil C, Fardoux J, Nguyen P, Marchetti R, Nouwen N, Molinaro A, Mergaert P, Giraud E. 2016. A peptidoglycan-remodeling enzyme is critical for bacteroid differentiation in *Bradyrhizobium* spp. during legume symbiosis. Mol Plant Microbe Interact 29:447–457. doi:10.1094/MPMI-03-16-0052-R. 26959836

[91] Khan SR, Gaines J, Roop RM, II, Farrand SK. 2008. Broad-host-range expression vectors with tightly regulated promoters and their use to examine the influence of TraR and TraM expression on Ti plasmid quorum sensing. Appl Environ Microbiol 74:5053–5062. doi:10.1128/AEM.01098-08. 18606801 PMC2519271

[92] Ducret A, Quardokus EM, Brun YV. 2016. MicrobeJ, a tool for high throughput bacterial cell detection and quantitative analysis. Nat Microbiol 1:16077. doi:10.1038/nmicrobiol.2016.77. 27572972 PMC5010025

[93] Schneider CA, Rasband WS, Eliceiri KW. 2012. NIH Image to ImageJ: 25 years of image analysis. Nat Methods 9:671–675. doi:10.1038/nmeth.2089. 22930834 PMC5554542

[94] Benincasa M, Barrière Q, Runti G, Pierre O, Bourge M, Scocchi M, Mergaert P. 2016. Single cell flow cytometry assay for peptide uptake by bacteria. Bio Protoc 6:e2038. doi:10.21769/BioProtoc.2038. PMC11915461 40110178

