# Supplementary material for: Sinorhizobium meliloti Functions Required for Resistance to Antimicrobial NCR Peptides and Bacteroid Differentiation
Source: mBio. 2021 Jul 27;12(4):e00895-21. doi: 10.1128/mBio.00895-21 (PMC8406287; doi:10.1128/mBio.00895-21)
Supplement: FIG S1 [file mbio.00895-21-sf001.pdf]

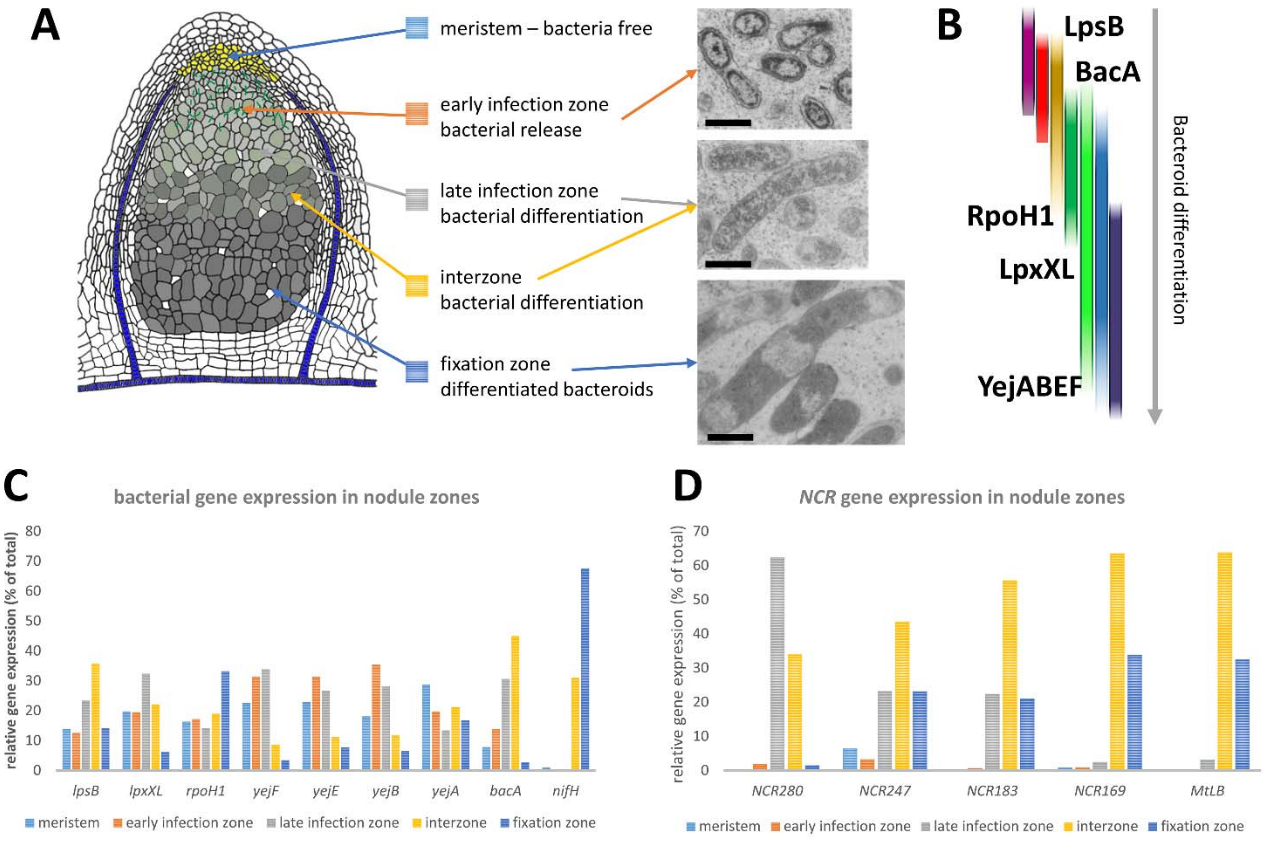

Figure S1. Expression pattern in nodules of bacterial and *NCR* genes. A. Schematic drawing of a *Medicago* nodule organized in functional zones. The meristem is bacteria-free and contains dividing cells allowing the organ to grow. In the early infection zone, bacteria proliferate in infection threads (green lines) and are released by endocytosis inside cells derived from the meristem. In the late infection zone and the so-called interzone, the bacteria differentiate into bacteroids. The fixation zone contains the fully differentiated, nitrogen-fixing bacteroids. The pictures show bacteria inside the nodule cells and are presented at the same scale (bar is 1 µm) allowing to appreciate the transformation of the bacteria. **B.** Against the backdrop of a changing landscape of NCR peptides (rainbow colors representing schematically peptides appearing and disappearing at different stages of symbiotic cell differentiation), the bacterial functions described in this study are critical at distinct stages of the bacteroid differentiation process. It should be noted that the functions that are essential in early stages, such as LpsB and BacA, can also be important in later stages of the bacteroid differentiation. However, the phenotypic analysis of the corresponding mutants cannot reveal these putative late roles. **C,D.** The relative expression profile (% of total) of the studied bacterial genes *lpsB*, *lpxXL*, *rpoH1*, *yejF*, *yejE*, *yejB*, *yejA* and *bacA* (**C**) and of *NCR280*, *NCR247*, *NCR183* and *NCR169* (**D**) in the meristem, early infection zone, late infection zone, interzone and fixation zone of *M. truncatula* nodules are displayed. The expression patterns of the *S. meliloti nifH* gene, encoding a nitrogenase subunit, and of the *M. truncatula* leghemoglobin genes (*MtLb*) are included in (**C**) and (**D**) respectively, as a reference indicating the completion of bacteroid formation and the onset of nitrogen fixation. Data was extracted from (1) and was obtained by RNA-seq analysis on laser-microdissected nodule tissues (2).

1. Guefrachi I, Nagymihaly M, Pislariu CI, Van de Velde W, Ratet P, Mars M, Udvardi MK, Kondorosi E, Mergaert P, Alunni B. 2014. Extreme specificity of *NCR* gene expression in *Medicago truncatula*. BMC Genom 15:712.
2. Roux B, Rodde N, Jardinaud M-F, Timmers T, Sauviac L, Cottret L, Carrère S, Sallet E, Courcelle E, Moreau S, Debellé F, Capela D, de Carvalho-Niebel F, Gouzy J, Bruand C, Gamas P. 2014. An integrated analysis of plant and bacterial gene expression in symbiotic root nodules using laser-capture microdissection coupled to RNA sequencing. Plant J 77:817–837.
