# Supplementary material for: Sinorhizobium meliloti Functions Required for Resistance to Antimicrobial NCR Peptides and Bacteroid Differentiation
Source: mBio. 2021 Jul 27;12(4):e00895-21. doi: 10.1128/mBio.00895-21 (PMC8406287; doi:10.1128/mBio.00895-21)
Supplement: TABLE S1 [file mbio.00895-21-st001.pdf]

Table S1. Strains and primers used for mutant constructions.

STRAINS

***Escherichia coli* strains**	**Relevant characteristics**	**Source**
DH5α.pVO155-p*npt2* ::*GFP*	Strain carrying the pVO155-p*npt2* ::*GFP* plasmid used for insertional mutagenesis	1
DH5α.pNPTS129	Strain carrying the pNPTS129 plasmid used for the construction of deletion mutants	1
DH5α.pSRKGm	Broad host-range expression vector derived from pBBR1	2
DH5α.pHC60-p*nifH* ::*GFP*	Strain used for triparental conjugation, carrying a derivative of the broad host range low copy number IncP plasmid pHC60 with a *GFP* gene under the control of the *nifH* promoter	Réné Geurts laboratory, Wageningen University
HB101.pRK600	Helper strain used for triparental conjugation	common laboratory strain

***Sinorhizobium meliloti* strains**	**Relevant characteristics**	**Source**
Sm1021	*Sinorhizobium meliloti* wild type strain	common laboratory strain
Sm1021.Δ*bacA*	Sm1021 derivative carrying a *bacA* deletion	3
Sm1021.*lpsB* ::Tn*5*	Sm1021 derivative carrying a Tn*5* transposon insertion in the *lpsB* gene	4
Sm1021.*lpxXL* ::pJH104	Sm1021 derivative carrying a plasmid insertion in *lpxXL*	5
Sm1021.*rpoH1* ::pVO155	Sm1021 derivative carrying a pVO155-p*npt2* ::*GFP* in the *rpoH1* gene	this work
Sm1021.*yejA* ::pVO155	Sm1021 derivative carrying a pVO155-p*npt2* ::*GFP* in the *yejA* gene	this work
Sm1021.*yejE* ::pVO155	Sm1021 derivative carrying a pVO155-p*npt2* ::*GFP* in the *yejE* gene	this work
Sm1021.*yejF* ::pVO155	Sm1021 derivative carrying a pVO155-p*npt2* ::*GFP* in the *yejF* gene	this work
Sm1021.Δ*yejA*	Sm1021 derivative carrying a *yejA* deletion	this work
Sm1021.Δ*yejE*	Sm1021 derivative carrying a *yejE* deletion	this work
Sm1021.Δ*yejF*	Sm1021 derivative carrying a *yejF* deletion	this work
Sm1021.pSRKGm	Sm1021 derivative carrying the empty pSRKGm plasmid	this work
Sm1021.pSRK-*yejABEF*	Sm1021 derivative carrying the *yejABEF* genes on the pSRKGm plasmid	this work
Sm1021.Δ*yejE* .pSRKGm	Sm1021.Δ*yejE* derivative carrying the empty pSRKGm plasmid	this work
Sm1021.Δ*yejE* .pSRK-*yejABEF*	Sm1021.Δ*yejE* derivative carrying the *yejABEF* genes on the pSRKGm plasmid	this work
Sm1021.Δ*yejF* .pSRKGm	Sm1021.Δ*yejF* derivative carrying the empty pSRKGm plasmid	this work
Sm1021.Δ*yejF* .pSRK-*yejABEF*	Sm1021.Δ*yejF* derivative carrying the *yejABEF* genes on the pSRKGm plasmid	this work
Sm1021.pHC60-p*nifH* ::*GFP*	Sm1021 carrying the pHC60-p*nifH* ::GFP plasmid	this work
Sm1021.Δ*bacA* .pHC60-p*nifH* ::GFP	Sm1021.Δ*bacA* carrying the pHC60-p*nifH* ::*GFP* plasmid	this work
Sm1021.Δ*yejE* .pHC60-p*nifH* ::*GFP*	Sm1021.Δ*yejE* carrying the pHC60-p*nifH* ::*GFP* plasmid	this work
Sm1021.Δ*yejF* .pHC60-p*nifH* ::*GFP*	Sm1021.Δ*yejF* carrying the pHC60-p*nifH* ::*GFP* plasmid	this work

PRIMERS

	**Targeted gene**	**Primer name**	**Sequence**	**Description**
Insertion mutants	SMc02829 (*yejF*)	2829forward	CAAATCGGTGTCGACCAACTCGATCCTGAAGCTC	Primers used to amplify a region of *SMc02829* CDS, flanked with SalI & XbaI sites
		2829reverse	GTTGGCTCTAGACATGGCTATCATTAC	
		2829out-f	CATCTCCTTCGACATCAAG	Primers used for verification
		2829out-r	CTCGTCGGCGATCAGGAGCTC	
	SMc02830 (*yejE*)	2830forward	CTTCGGCCTGTCGACGTTTGCCGAGTTCATC	Primers used to amplify a region of *SMc02830* CDS, flanked with SalI & XbaI sites
		2830reverse	CAGCGCTCTAGAGAAAAGGACCGAGATG	
		2830out-f	CTGGCTCTTCCTCCTCCTCTTC	Primers used for verification
		2830out-r	GATGATGAGCAGGATGTAGAG	
	SMc02832 (*yejA*)	2832forward	GCTACGTCGACGATCGGAGAACCCAAATAC	Primers used to amplify a region of *SMc02832* CDS, flanked with SalI & XbaI sites
		2832reverse	GTGACTCTAGACTCGCCTGTCTTTTC	
		2832out-f	CAATGCCGAGGAACAACC	Primers used for verification
		2832out-r	GAGAATGTTCGGCAGTTC	
	SMc00646 (*rpoH1*)	RpoH1-Sal1-forward	CGCACAGTCGACTAACGAGCCATCTTCGCCTC	Primers used to amplify a region of *SMc00646* CDS, flanked with SalI & XbaI sites
		RpoH1-Xba1-Reverse	CGAAGATCTAGAGCTCACGATCGTTCAGAACC	
		RpoH1-up	AAGCGGTATCAGGAG	Primers used for verification
		RpoH1-down	CCTCCTGCACCTTTT	

PRIMERS (cont.)

	Targeted gene	Primer name	Sequence	Description
Deletion mutants	SMc02829 (*yejF*)	2829fup	GCAACGGCACCATCATGGATCCGCACC	Up fragment used for deletion of *SMc02829*
		2829rup	GGAGAAACTCGGAACGTCTTTCTCG	
		2829fdown	CGTTCCGAGTTTCTCCCATGC	Down fragment used for deletion of *SMc02829*
		2829rdown	CACCCACTCGCTCATCCAAGCTTTCAGG	
	SMc02830 (*yejE*)	2830fup	GGACACGTGTTCCGCAACGGATCCCTGATC	Up fragment used for deletion of *SMc02830*
		2830rup	CGTCGCGCCAGACGTCCCTCCG	
		2830fdown	GGACGTCTGGCGCGACGATGACAG	Down fragment used for deletion of *SMc02830*
		2830rdown	GCGAACATGCCTGAAAGCTTCGTCG	
	SMc02832 (*yejA*)	2832fup	GGTCGAACCGCAGGGATCCAGCGCAGCACG	Up fragment used for deletion of *SMc02832*
		2832rup	GCAAGAGCTCCGGCCGGTACCCC	
		2832fdown	CGGCCGGAGCTCTTGCGCTCAGG	Down fragment used for deletion of *SMc02832*
		2832rdown	CATGATGCCGACGATCGTCAAGCTTATCAGC	
comple-mentation	*yejABEF*	yejA_NdeI_F	ATTATAATCATATGCCAAACTTCTGCAGGACCG	upstream primer for cloning of yejABEF in pSRK(Gm)
		yejF_XbaI_R	ATAATTATCTAGACTACTGATGAACGGCGGC	downstream primer for cloning of yejABEF in pSRK(Gm)
		LacI_F	ACGACAGGTTTCCCGACTGG	primer for construct verification in pSRK(Gm)
		T7_F	TAATACGACTCACTATAGGG	primer for construct verification in pSRK(Gm)

References

1 Gully D, Gargani D, Bonaldi K, Grangeteau C, Chaintreuil C, Fardoux J, Nguyen P, Marchetti R, Nouwen N, Molinaro A, Mergaert P, Giraud E. 2016. A peptidoglycan-remodeling enzyme is critical for bacteroid differentiation in Bradyrhizobium spp. during legume symbiosis. Mol Plant-Microbe Interact 29:447–457.

2 Khan SR, Gaines J, Roop RM 2nd, Farrand SK. 2008. Broad-host-range expression vectors with tightly regulated promoters and their use to examine the influence of TraR and TraM expression on Ti plasmid quorum sensing. Appl Environ Microbiol 74:5053-5062.

3 Haag AF, Baloban M, Sani M, Kerscher B, Pierre O, Farkas A, Longhi R, Boncompagni E, Hérouart D, Dall'angelo S, Kondorosi E, Zanda M, Mergaert P, Ferguson GP. 2011. Protection of *Sinorhizobium* against host cysteine-rich antimicrobial peptides is critical for symbiosis. PLoS Biol 9:e1001169.

4 Campbell GRO, Sharypova LA, Scheidle H, Jones KM, Niehaus K, Becker A, Walker GC. 2003. Striking complexity of lipopolysaccharide defects in a collection of *Sinorhizobium meliloti* mutants. J Bacteriol 185:3853–3862.

5 Haag AF, Wehmeier S, Beck S, Marlow VL, Fletcher V, James EK, Ferguson GP. 2009. The *Sinorhizobium meliloti* LpxXL and AcpXL proteins play important roles in bacteroid development within alfalfa. J Bacteriol 191:4681–4686.
