# Supplementary material for: Sinorhizobium meliloti Functions Required for Resistance to Antimicrobial NCR Peptides and Bacteroid Differentiation
Source: mBio. 2021 Jul 27;12(4):e00895-21. doi: 10.1128/mBio.00895-21 (PMC8406287; doi:10.1128/mBio.00895-21)
Supplement: TABLE S2 [file mbio.00895-21-st002.pdf]

Table S2. Survival after NCR treatment of wild-type and mutant *Sinorhizobium meliloti* .

peptide (concentration)	survival (%) relative to untreated bacteria (n=3)	Standard deviation	survival relative to WT (%)	*p* -value (Dunn test)
**NCR169 (10 µM)**				
WT	8,73	3,41	100,00	
bacA	4,80	4,43	55,00	0,05
yejA	10,91	8,52	125,00	0,30
yejE	9,45	1,70	108,33	0,26
yejF	13,67	8,86	156,67	0,23
lpxXL	1,02	0,34	11,67	0,02
lpsB	6,55	1,70	75,00	0,48
rpoH	7,56	0,68	86,67	0,23
**NCR183 (10 µM)**				
WT	0,05	0,03	100,00	
bacA	0,01	0,01	12,50	0,24
yejA	0,13	0,11	275,00	0,27
yejE	0,00	0,00	0,00	0,02
yejF	0,00	0,00	0,00	0,02
lpxXL	0,00	0,00	0,00	0,02
lpsB	0,00	0,01	5,00	0,12
rpoH	0,23	0,15	487,50	0,15
**NCR247 (25 µM)**				
WT	0,03	0,02	100,00	
bacA	0,00	0,00	3,85	0,08
yejA	0,05	0,00	153,85	0,37
yejE	0,00	0,00	3,85	0,03
yejF	0,00	0,00	0,00	0,01
lpxXL	0,00	0,00	0,00	0,01
lpsB	0,00	0,00	0,00	0,01
rpoH	0,00	0,00	0,00	0,01
**NCR280 (25 µM)**				
WT	0,05	0,01	100,00	
bacA	0,01	0,02	23,91	0,23
yejA	0,00	0,00	2,17	0,02
yejE	0,00	0,00	0,00	0,01
yejF	0,01	0,01	10,87	0,14
lpxXL	0,00	0,00	0,00	0,01
lpsB	0,00	0,00	0,00	0,01
rpoH	0,07	0,02	134,78	0,29
**NCR247 (25 µM)**				
WT.pSRK-Gm	0,16	0,04	100,00	
yejF .pSRK-Gm	0,00	0,00	0,12	0,01
WT.pSRK-*yejABEF*	0,14	0,04	87,99	0,36
yejF .pSRK-*yejABEF*	0,19	0,08	117,20	0,36
