# Supplementary material for: Sinorhizobium meliloti Functions Required for Resistance to Antimicrobial NCR Peptides and Bacteroid Differentiation
Source: mBio. 2021 Jul 27;12(4):e00895-21. doi: 10.1128/mBio.00895-21 (PMC8406287; doi:10.1128/mBio.00895-21)
Supplement: FIG S2 [file mbio.00895-21-sf002.pdf]

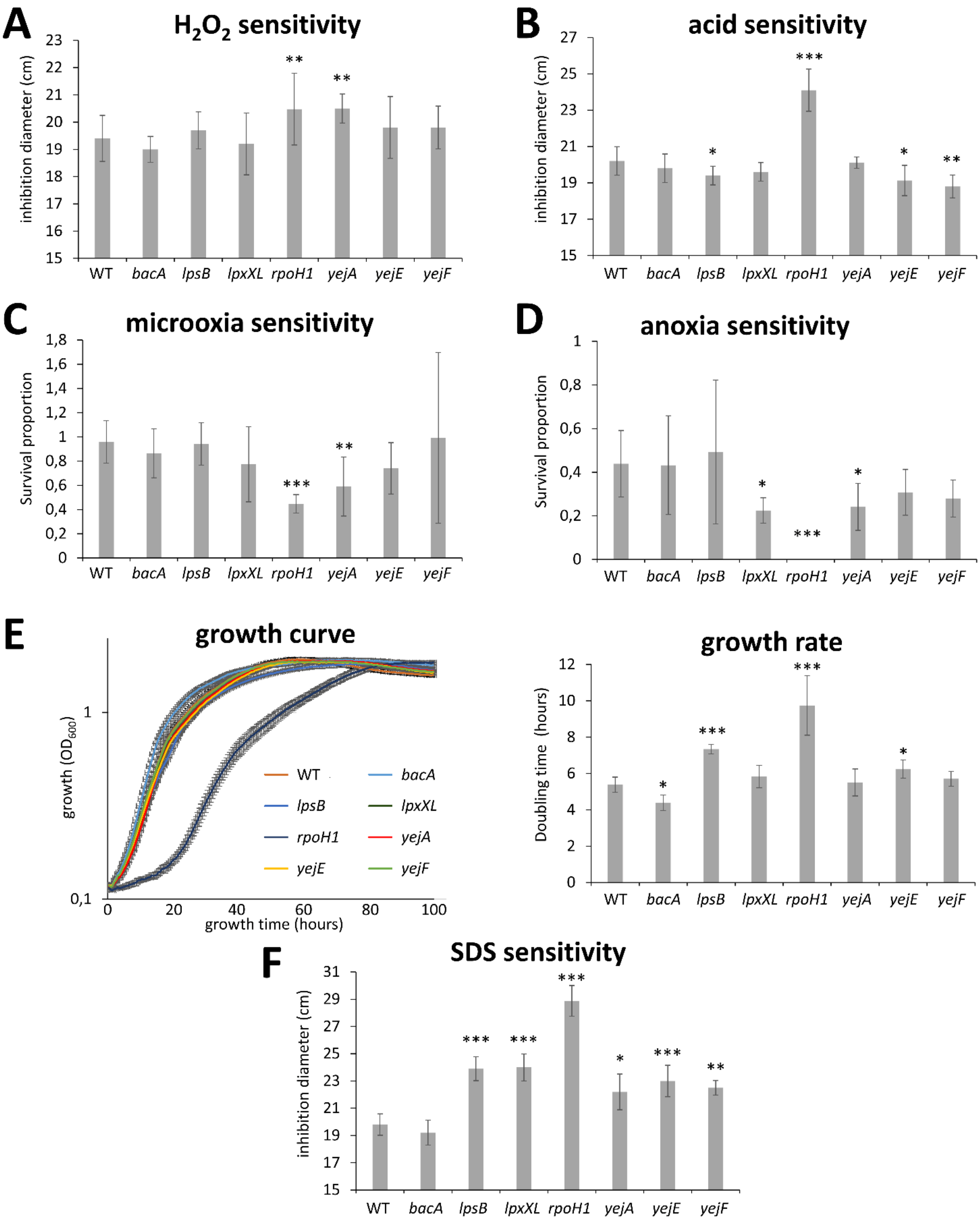

Figure S2. Phenotypes of *Sinorhizobium meliloti* mutants in *in vitro* stress conditions. A. Growth inhibition in the presence of H_2O_2 (n=10). **B.** Growth inhibition in the presence of HCl (n=10). **C.** Growth inhibition in microoxia (n=4). **D.** Growth inhibition in anoxia (n=4). **E.** Growth curves and growth rate in liquid medium (n=10). **F.** Growth inhibition in the presence of SDS (n=10). Growth inhibition in (**A**,**B**,**F**) was determined by the size of a halo formed around a paper patch soaked in the stress compound and in (**C**,**D**) by colony forming units counting. Error bars in all panels are standard deviations. Nonparametric Kruskal-Wallis and post-hoc Dunn tests were used to assess the significance of differences in the data of panels A to F (* is $p<0.05$; ** is $p<0.01$; *** is $p<0.001$). One representative experiment out of at least two is shown.
