# Supplementary material for: Sinorhizobium meliloti Functions Required for Resistance to Antimicrobial NCR Peptides and Bacteroid Differentiation
Source: mBio. 2021 Jul 27;12(4):e00895-21. doi: 10.1128/mBio.00895-21 (PMC8406287; doi:10.1128/mBio.00895-21)
Supplement: FIG S3 [file mbio.00895-21-sf003.pdf]

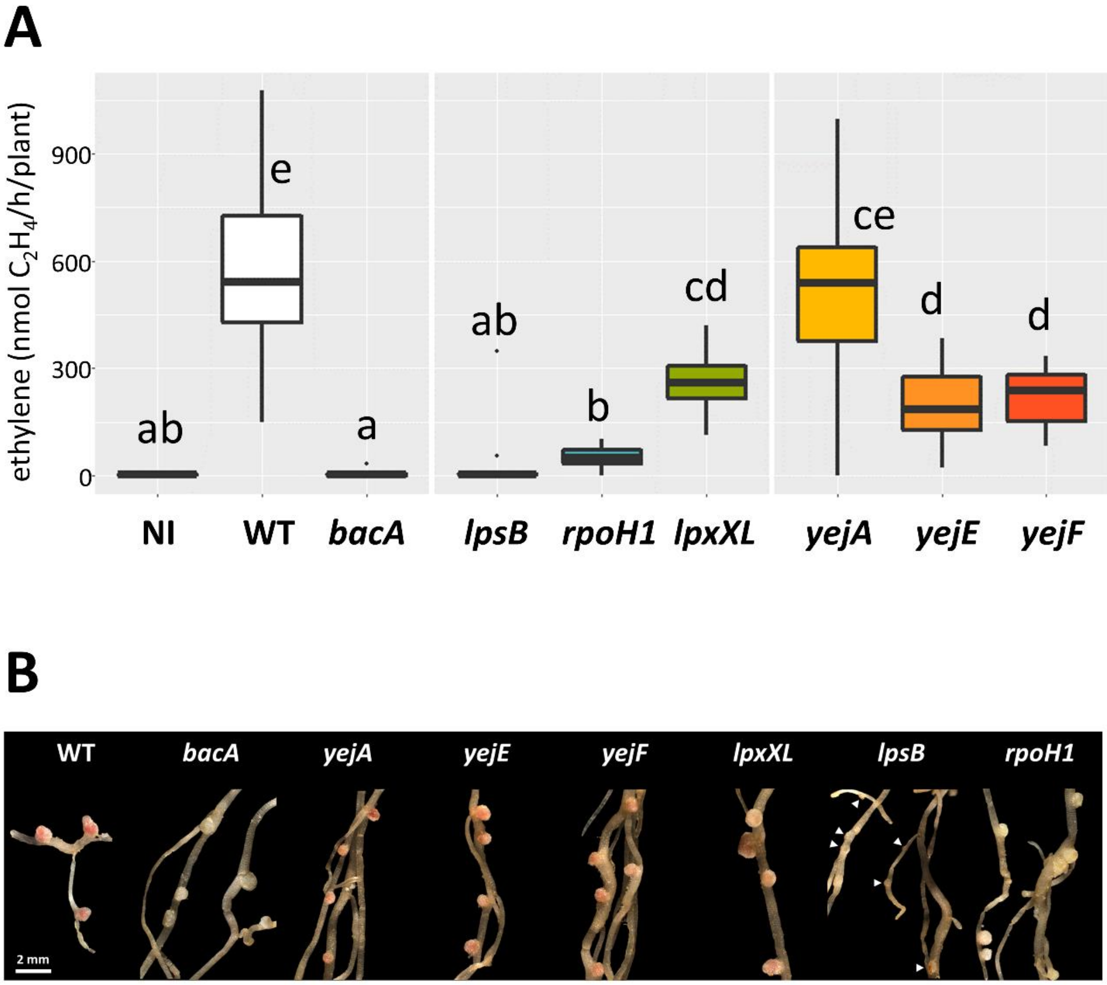

Figure S3. Symbiotic phenotypes of *Sinorhizobium meliloti* NCR-sensitive mutants in *Medicago truncatula*. A. Nitrogen fixation activity determined by the acetylene reduction assay on whole roots of nodulated plants infected with the indicated bacterial mutants at 21 days post inoculation. NI, non-inoculated control plants; WT, plants nodulated by the wild-type strain Sm1021. Boxplots were generated from 15 plants each. Letters associated with each condition represent statistically different classes determined by a non-parametric Dunn test, with a α threshold equal to 0.05. **B.** Nodule phenotypes at 21 days post inoculation. Arrowheads indicate small nodules elicited by the *lpsB* mutant. Scale bar (2 mm) applies to all panels.
