# Supplementary material for: Sinorhizobium meliloti Functions Required for Resistance to Antimicrobial NCR Peptides and Bacteroid Differentiation
Source: mBio. 2021 Jul 27;12(4):e00895-21. doi: 10.1128/mBio.00895-21 (PMC8406287; doi:10.1128/mBio.00895-21)
Supplement: FIG S4 [file mbio.00895-21-sf004.pdf]

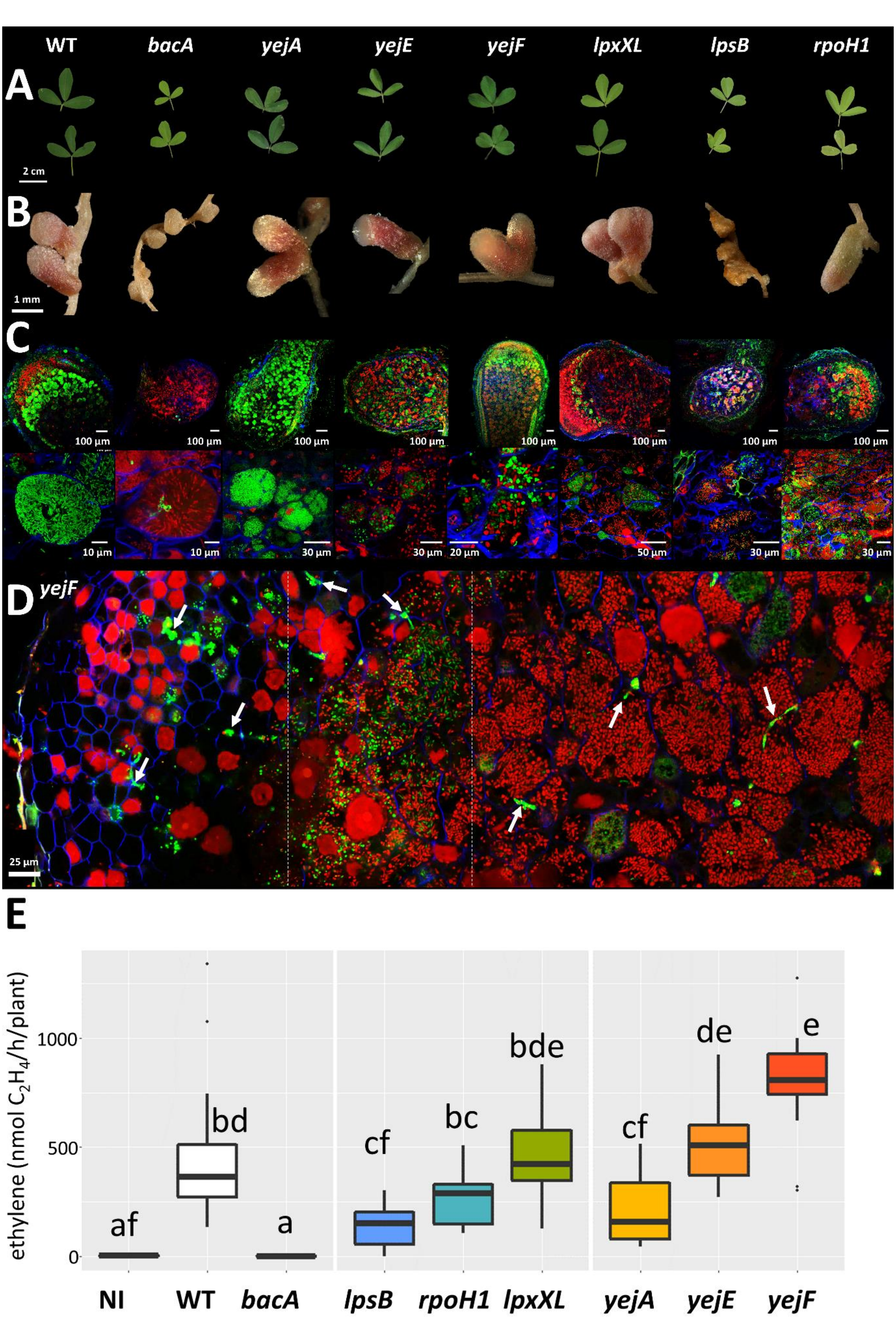

WT
bacA
yejA
yejE
yejF
lpxXL
lpsB
rpoH1
A
2 cm
B
1 mm
C
100 µm
10 µm
30 µm
20 µm
50 µm
D
yejF
25 µm
E
ethylene (nmol C_2H_4/h/plant)
1000
500
0
af
bd
a
cf
bc
bde
cf
de
e
NI
WT
bacA
lpsB
rpoH1
lpxXL
yejA
yejE
yejF

Figure S4. Symbiotic phenotype of *Sinorhizobium meliloti* mutants during symbiosis with *Medicago sativa*. A. Leaves of plants inoculated with the indicated bacterial strains. Scale bar = 2 cm. **B.** Nodule phenotype at 21 days post inoculation. Scale bar = 1 mm. **C.** Bacteroid viability determined by live-dead staining of nodule sections and confocal microscopy. Top row images, full nodule sections; Bottom row images, enlarged images of symbiotic cells. Scale bars are indicated in each panel. **D.** Composite image of a *yejF*-infected nodule section showing the rapid permeabilization of the membranes of the internalized bacteria (red staining). Note the bacteria in infection threads (white arrows) are not permeabilized (green staining). The images of the composition are separated by dashed lines. **E.** Nitrogen fixation activity determined by the acetylene reduction assay on whole roots of nodulated plants infected with the indicated bacterial mutants at 21 days post inoculation. NI, non-inoculated control plants; WT, plants nodulated by the wild-type strain Sm1021. Boxplots were generated from 15 plants each. Letters associated with each condition represent statistically different classes determined by a non-parametric Dunn test, with a α threshold equal to 0.05.
