# Supplementary material for: Sinorhizobium meliloti Functions Required for Resistance to Antimicrobial NCR Peptides and Bacteroid Differentiation
Source: mBio. 2021 Jul 27;12(4):e00895-21. doi: 10.1128/mBio.00895-21 (PMC8406287; doi:10.1128/mBio.00895-21)
Supplement: FIG S5 [file mbio.00895-21-sf005.pdf]

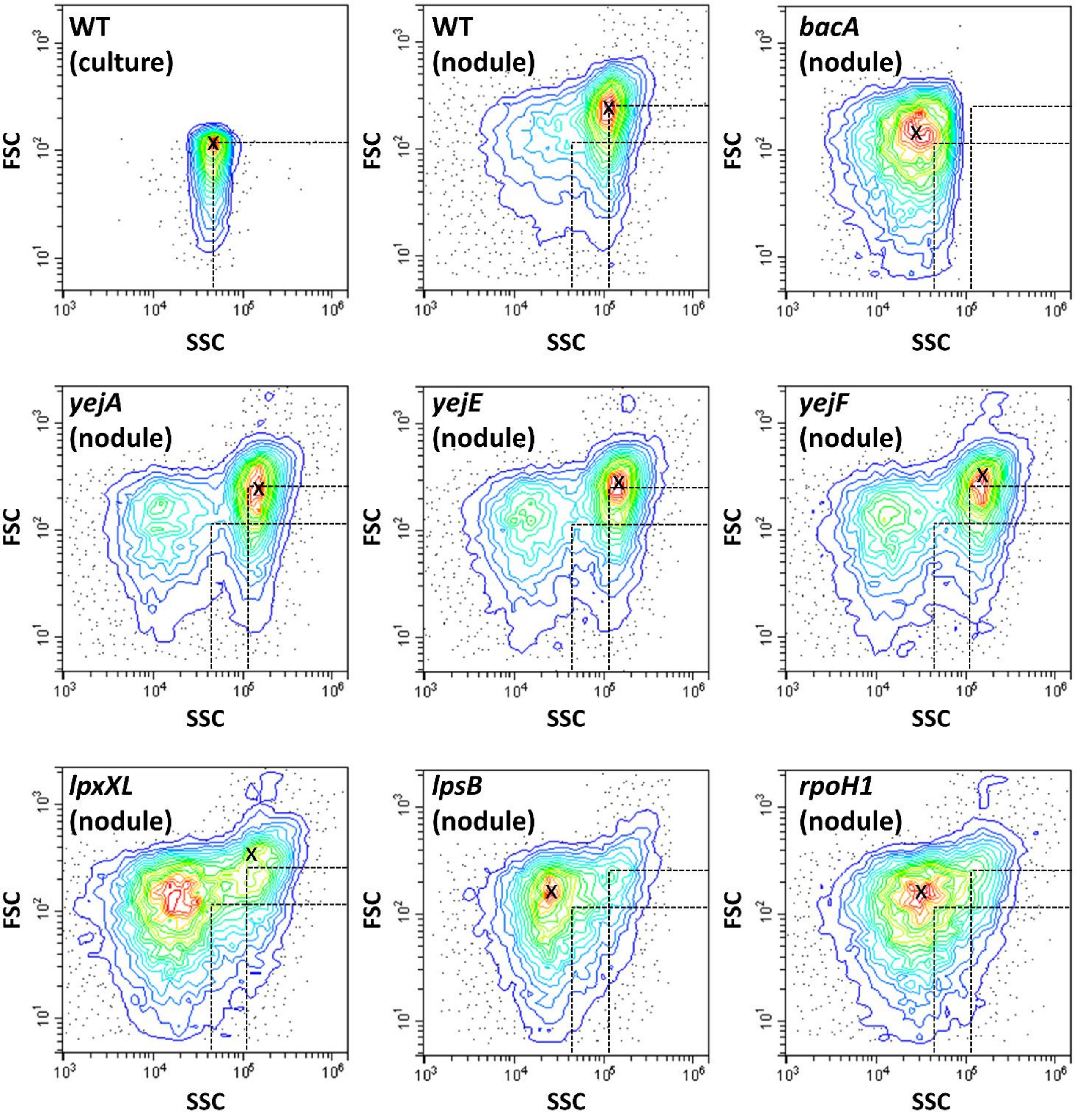


**Figure S5. Morphological parameters in nodule bacteria of *Medicago truncatula* nodules.** Flow cytometry analysis of the morphology of bacteria isolated from nodules infected with the indicated strains. The forward scatter (FSC, y-axes) is represented in function of the side scatter (SSC, x-axes). The "X" indicates the peak values for each sample. The position of the peak value in the cultured bacteria and in the wild-type bacteroids are indicated in each panel by the hatched lines.
