# Supplementary material for: Sinorhizobium meliloti Functions Required for Resistance to Antimicrobial NCR Peptides and Bacteroid Differentiation
Source: mBio. 2021 Jul 27;12(4):e00895-21. doi: 10.1128/mBio.00895-21 (PMC8406287; doi:10.1128/mBio.00895-21)
Supplement: FIG S6 [file mbio.00895-21-sf006.pdf]

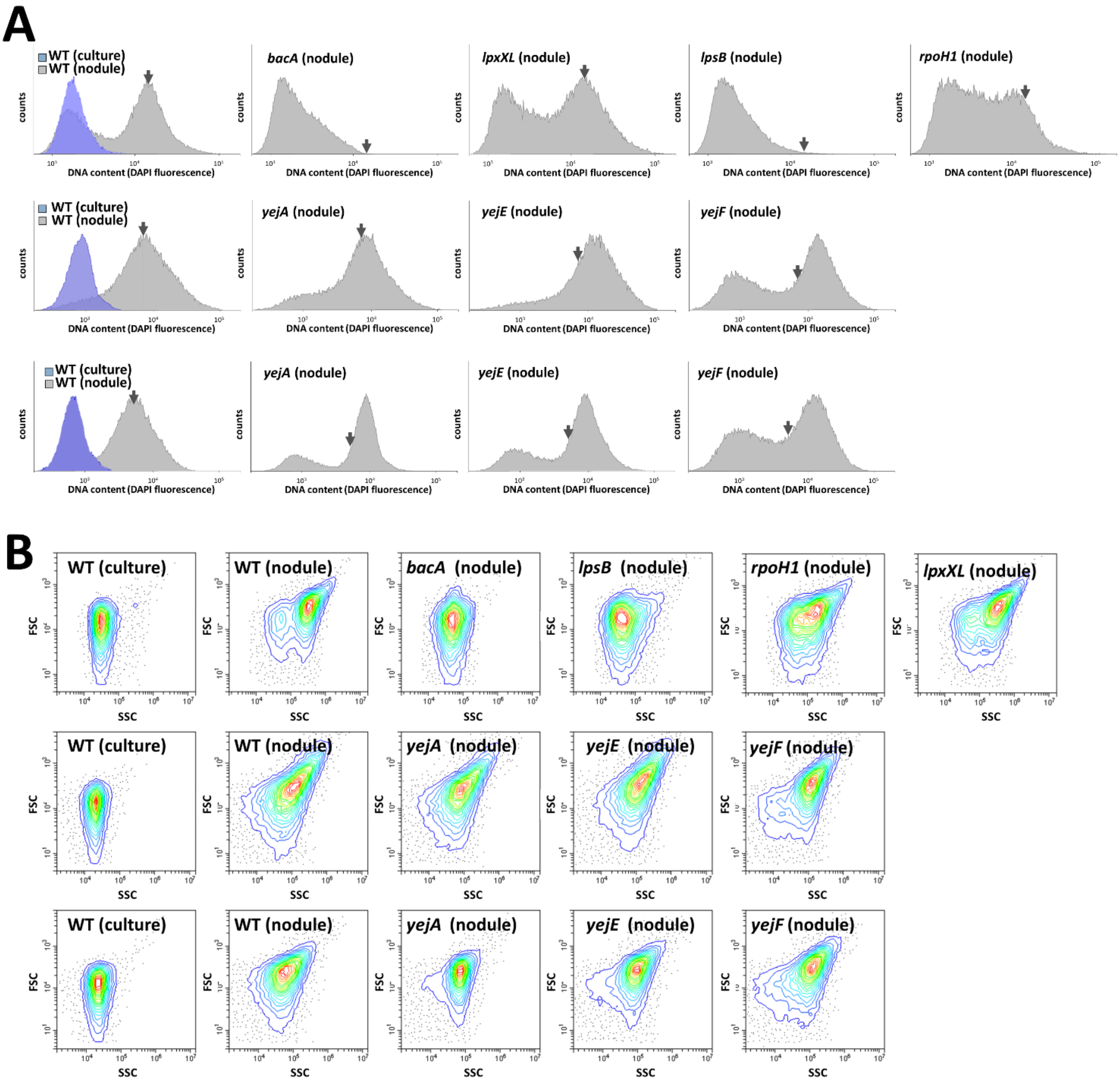

Figure S6. DNA content and morphological parameters in nodule bacteria in *Medicago sativa*. A. Flow cytometry analysis of the DNA content of bacteria in culture or isolated from nodules infected with the indicated strains and stained with 4',6-diamidino-2-phenylindole (DAPI). The cell counts (y-axes) are represented in function of the DAPI fluorescence (x-axes). The arrow in each panel indicates the mean DNA content of wild-type bacteroids. The rows represent three sets of independent experiments and the values of DAPI fluorescence cannot be compared between experiments because of differences in instrument settings. The first two rows are samples prepared from 21 dpi nodules; the third row is from 32 dpi nodules. The latter shows that the phenotype of the *yejA* mutant evolves in older nodules. **B.** Flow cytometry analysis of the morphology of bacteria isolated from nodules infected with the indicated strains. The forward scatter (FSC, y-axes) is represented in function of the side scatter (SSC, x-axes).
