# Supplementary material for: Sinorhizobium meliloti Functions Required for Resistance to Antimicrobial NCR Peptides and Bacteroid Differentiation
Source: mBio. 2021 Jul 27;12(4):e00895-21. doi: 10.1128/mBio.00895-21 (PMC8406287; doi:10.1128/mBio.00895-21)
Supplement: FIG S7 [file mbio.00895-21-sf007.pdf]

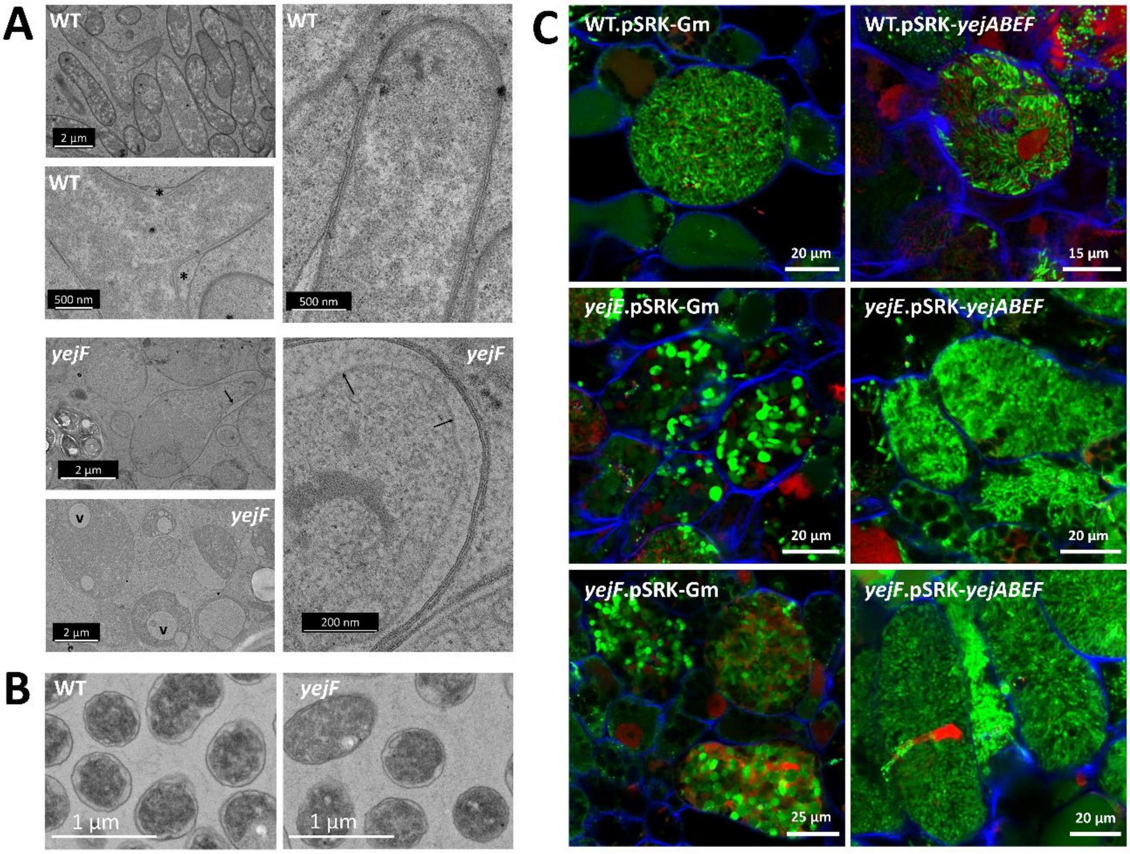

Figure S7. Cellular defects in the *yejF* mutant bacteroids in *Medicago sativa* nodules. A. Transmission electron microscopy of wild type (WT) and *yejF* mutant bacteroids. The arrows indicate retracted inner membranes in the *yejF* bacteroids, "v" indicate vacuoles and "*" indicate an enlarged peribacteroid space found in the angle of a branched bacteroid. **B.** Ultrastructure of wild type (WT) and the *yejF* mutant bacteria in culture by transmission electron microscopy. Scale bars are 1 µm. **C.** Confocal microscopy of sections of nodules infected with wild type (WT), *yejE* or *yejF* mutant bacteria carrying the empty plasmid pSRK-Gm or the complementing plasmid pSRK-*yejABEF*.
