# Supplementary material for: Sinorhizobium meliloti Functions Required for Resistance to Antimicrobial NCR Peptides and Bacteroid Differentiation
Source: mBio. 2021 Jul 27;12(4):e00895-21. doi: 10.1128/mBio.00895-21 (PMC8406287; doi:10.1128/mBio.00895-21)
Supplement: FIG S8 [file mbio.00895-21-sf008.pdf]

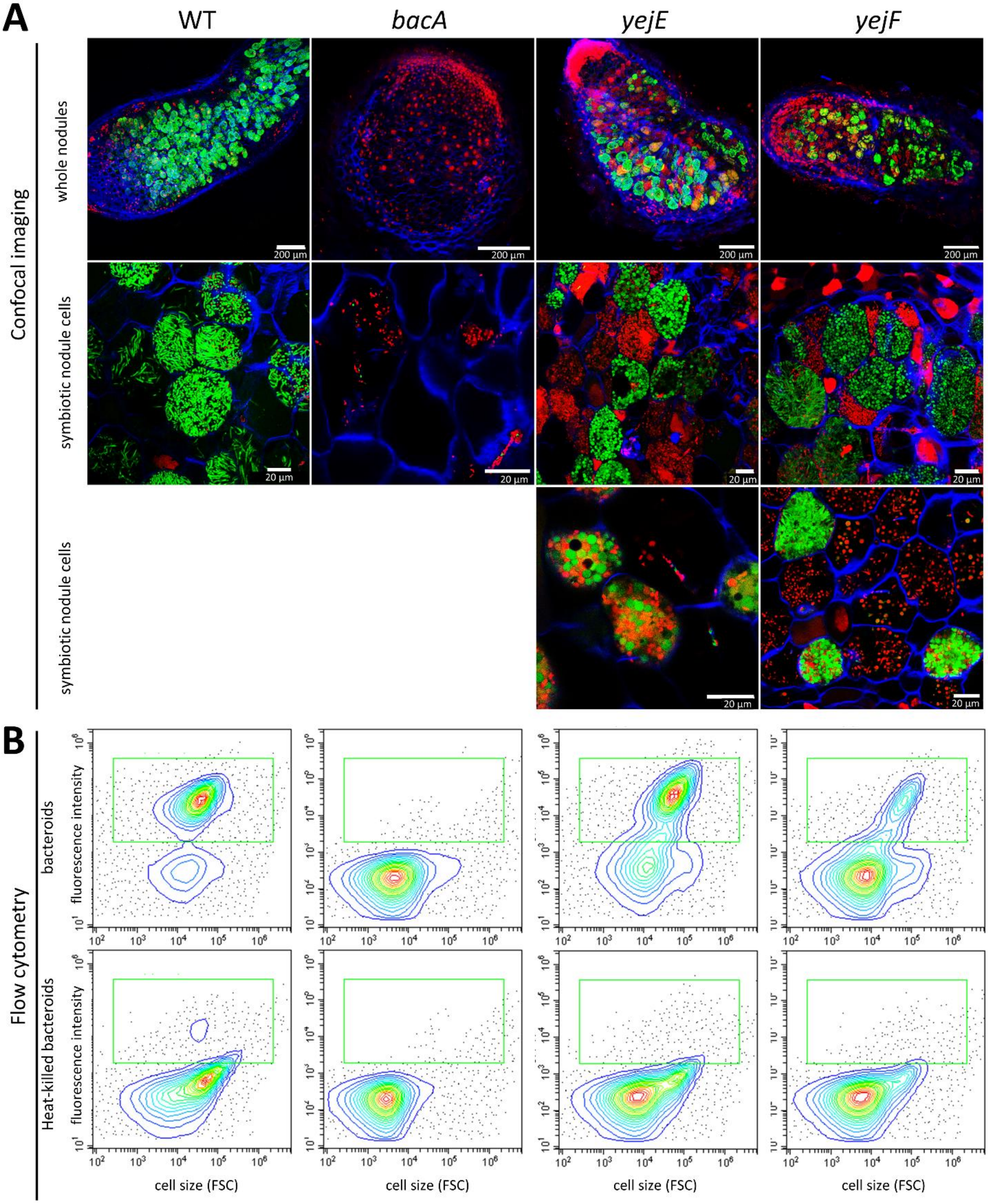

Figure S8. Nitrogenase expression in the *yejE* and *yejF* mutant bacteroids in *Medicago sativa* nodules. A. Confocal microscopy of sections of nodules infected with *S. meliloti* Sm1021.pHC60-p*nifH*::*GFP* (WT), Sm1021.Δ*bacA*.pHC60-p*nifH*::*GFP* (*bacA*), Sm1021.Δ*yejE*.pHC60-p*nifH*::*GFP* (*yejE*) or Sm1021.Δ*yejF*.pHC60-p*nifH*::*GFP* (*yejF*) and stained with propidium iodide (red stain). Green stained bacteroids are functional while red stained bacteroids are non-functional. **B.** Flow cytometry determination of GFP levels in nodule bacteria (upper panels) and heat-killed nodule bacteria (lower panels). The green square shows the position of the GFP-positive bacteroids. FSC is forward scatter.
